# Differences between subclinical attention-deficit/hyperactivity and autistic traits in default mode, salience, and frontoparietal network connectivities in young adult Japanese

**DOI:** 10.1038/s41598-023-47034-7

**Published:** 2023-11-13

**Authors:** Risa Hirata, Sayaka Yoshimura, Key Kobayashi, Morio Aki, Mami Shibata, Tsukasa Ueno, Takashi Miyagi, Naoya Oishi, Toshiya Murai, Hironobu Fujiwara

**Affiliations:** 1 Department of Neuropsychiatry, Kyoto University Hospital, 54 Shogoinkawaracho, Sakyo-ku, Kyoto, 6068397 Japan; 2 Faculty of Human Health Science, Graduate School of Medicine, Kyoto University, Kyoto, Japan; 3 Organization for Promotion of Neurodevelopmental Disorder Research, Kyoto, Japan; 4 Department of Neuropsychiatry, Graduate School of Medicine, University of Kyoto, Kyoto, Japan; 5 Integrated Clinical Education Center, Kyoto University Hospital, Kyoto, Japan; 6 Medical Innovation Center, Kyoto University Graduate School of Medicine, Kyoto, Japan; 7 Artificial Intelligence Ethics and Society Team, RIKEN Center for Advanced Intelligence Project, Tokyo, Japan; 8 The General Research Division, Osaka University Research Center on Ethical, Legal and Social Issues, Kyoto, Japan

**Keywords:** ADHD, Autism spectrum disorders, Cognitive neuroscience

## Abstract

Attention-deficit hyperactivity disorder (ADHD) and autism spectrum disorder (ASD) are associated with attentional impairments, with both commonalities and differences in the nature of their attention deficits. This study aimed to investigate the neural correlates of ADHD and ASD traits in healthy individuals, focusing on the functional connectivity (FC) of attention-related large-scale brain networks (LSBNs). The participants were 61 healthy individuals (30 men; age, 21.9 ± 1.9 years). The Adult ADHD Self-Report Scale (ASRS) and Autism Spectrum Quotient (AQ) were administered as indicators of ADHD and ASD traits, respectively. Performance in the continuous performance test (CPT) was used as a behavioural measure of sustained attentional function. Functional magnetic resonance imaging scans were performed during the resting state (Rest) and auditory oddball task (Odd). Considering the critical role in attention processing, we focused our analyses on the default mode (DMN), frontoparietal (FPN), and salience (SN) networks. Region of interest (ROI)-to-ROI analyses (false discovery rate < 0.05) were performed to determine relationships between psychological measures with within-network FC (DMN, FPN, and SN) as well as with between-network FC (DMN-FPN, DMN-SN, and FPN-SN). ASRS scores, but not AQ scores, were correlated with less frequent commission errors and shorter reaction times in the CPT. During Odd, significant positive correlations with ASRS were demonstrated in multiple FCs within DMN, while significant positive correlations with AQ were demonstrated in multiple FCs within FPN. AQs were negatively correlated with FPN-SN FCs. During Rest, AQs were negatively and positively correlated with one FC within the SN and multiple FCs between the DMN and SN, respectively. These findings of the ROI-to-ROI analysis were only partially replicated in a split-half replication analysis, a replication analysis with open-access data sets, and a replication analysis with a structure-based atlas. The better CPT performance by individuals with subclinical ADHD traits suggests positive effects of these traits on sustained attention. Differential associations between LSBN FCs and ASD/ADHD traits corroborate the notion of differences in sustained and selective attention between clinical ADHD and ASD.

Attention-deficit hyperactivity disorder (ADHD) and autism spectrum disorder (ASD) are neurodevelopmental disorders; ADHD is characterized by inattention and/or hyperactivity–impulsivity, whereas ASD is characterized by deficits in social communication and interaction, repetitive behaviours, and restricted interests. Both ASD and ADHD can cause attention deficits, and inattention and its associated problems are often reported in both disorders^1,2^. In clinical settings, it is often difficult to determine whether the deficits originate from ADHD or ASD; nevertheless, evidence suggests a difference in attentional processing between the two disorders. Several behavioural studies using neuropsychological tests have suggested that patients with ADHD experience problems with sustained attention, whereas those with ASD experience problems with selective attention^3–5^. Clarifying the mechanisms underlying these problems would lead to a better understanding of the disorders. However, it is unclear how the neural correlates of attentional processing differ between ADHD and ASD.

Recent neuroimaging studies using functional MRI (fMRI) have suggested that, in cognitive deficits and neuropsychiatric disorders, distributed circuit abnormalities can be characterized by changes in synchronized brain activities (i.e. functional connectivity [FC]). More specifically, resting-state fMRI (rs-fMRI) studies have revealed several networks that are consistently found in healthy people and represent specific patterns of synchronous activity; these networks are typically termed large-scale brain networks (LSBNs)^6,7^. Regarding LSBNs, the triple network model hypothesis concerns the interactions among three networks in particular—the salience network (SN), default mode network (DMN), and frontoparietal network (FPN)^8–11^ are often coactivated or deactivated during attentionally demanding tasks^12,13^. The SN detects the importance of internal or external stimuli and switches between networks^14,15^. The DMN is active during the resting state, such as passively viewing a stimulus; however, it is also active during social cognition and self-related processing^16–19^. It involves cognitive processes unrelated to the current situation (i.e. mind-wandering). In contrast to the DMN, the FPN is active during information processing and is important for maintaining and manipulating working memory, rule-based problem solving, and decision-making in goal-directed behaviour^20–23^.

Regarding these three LSBNs, previous studies have reported within-network as well as between-network FC abnormalities in psychiatric disorders, including ADHD and ASD. As for within-network connectivity, mixed findings were reported in within-SN FCs in ASD, with some showing hypoconnectivity and others showing hyperconnectivity^9,24–26^. In one study in ADHD, changes in within-SN FCs were not demonstrated during the resting state^27^. Within-DMN FC dysfunction has been consistently identified in ADHD, with no observed changes in within-DMN FCs in ASD^28,29^. Within-FPN FC hypoconnectivity during the resting state has been observed in children diagnosed with ADHD^30,31^, whereas no alterations were found in within-FPN FCs in adolescents with ASD^32^. Concerning inter-network connectivity, SN-FPN FC hyperconnectivity during the resting state has been associated with ADHD traits^33,34^, while hypoconnectivity has been noted in children with ASD^35,36^. SN-DMN FC hypoconnectivity during the resting state has been documented in adults with ADHD^37^, and hyperconnectivity has been reported in adults with ASD^38^. DMN-FPN FC hyperconnectivity during the resting state has been identified in both children and adults with ADHD^39,40^ as well as in adolescents with ASD^41^.

Subclinical traits of ADHD and ASD are continuously distributed throughout the general population^42^. Investigating the differences in the neural correlates of attentional processing between the subclinical traits of ADHD and ASD would provide deeper insights into these disorders and help establish interventional strategies in people with potential ADHD/ASD traits. This study aimed to investigate the neural correlates of ADHD and ASD traits in attentional processing among healthy individuals by evaluating the FCs of LSBNs using fMRI. We hypothesized that the subclinical traits of ADHD and ASD would have differential neural correlates in relation to the FCs within and between the SN, FPN, and DMN.

## Methods

### Participants

The participants were 61 healthy individuals (30 men; age, 21.9 ± 1.9 years; all right-handed). Two well-trained psychiatrists (KK and MS) confirmed that no participant had any psychiatric disorder or severe medical or neurological illness. The intelligence quotient of each participant was estimated using the Japanese version of the Adult Reading Test^43^, and all participants fell within the normal range. The experimental procedures were fully explained, and all participants provided written informed consent before participating in the study. The study was approved by the ethics committee of the Kyoto University Graduate School and Faculty of Medicine and adhered to the guidelines of the Declaration of Helsinki.

### Questionnaire to evaluate psychological status

The Japanese version of the Adult ADHD Self-Report Scale (ASRS) and the Autism Spectrum Quotient (AQ) were administered, and the scores were recorded as indicators of ADHD and ASD, respectively. The validity of the Japanese version of the ASRS has been confirmed previously based on the Japanese version of Conners’ Adult ADHD Rating Scales–Self Report subscales (0.59 ≤ *r ≤ *0.77) and the Beck Depression Inventory-II (*r *= 0.38), and its internal consistency and reliability has also been confirmed (Cronbach’s *α* approximately 0.80)^44–46^. The ASRS consists of two questionnaires—Part A (6 questions) and Part B (12 questions)—and the answers are recorded in terms of frequency on a 5-point scale with the following options: *never*, *rarely*, *sometimes*, *frequently*, and *very frequently*. Higher ASRS scores indicate higher levels of ADHD traits. The original Part A and Part B questionnaires are formatted with dark-shaded boxes such that endorsements in the darkly shaded boxes signify more severe symptoms. If a respondent marks ≥ 4 responses in the dark-shaded boxes in Part A, they are screened as positive for ADHD (that is, their current symptom profile is considered to be highly consistent with the diagnosis of ADHD)^47,48^.

The Japanese version of the AQ has good validity, as confirmed by a comparative study between a group with Asperger’s syndrome (AS) or high-functioning autism (HFA) and two control groups (a group of healthy adults and a group of university students). Tukey's multiple comparison test revealed that the AS/HFA group scored higher (*p *< 0.0001) on the AQ than the two control groups. The adults with AS/HFA had a mean AQ of 37.9, which was significantly higher than those of the two control groups (mean AQ score = 18.5 and 20.7). Moreover, 88% of the adults with AS/HFA scored > 33 points, whereas only 3% of participants in the two control groups scored > 33 points. The same study also confirmed the internal consistency and reliability of the AQ scale (Cronbach’s* a* = 0.81)^49^. In addition, the reliability and validity of this questionnaire have been tested and confirmed in several studies from various countries^50–52^. The AQ consists of 50 items that are rated by participants on a 4-point scale with the following options: *definitely agree*, *slightly agree*, *slightly disagree*, and *definitely disagree*. The 50 items are divided into five subscales (Social Skill, Attention Switching, Attention to Detail, Communication, and Imagination) consisting of 10 items each. The AQ score ranges from 0 to 50, and higher AQ scores indicate higher levels of autistic traits.

### Assessment of attentional function

We conducted the continuous performance test (CPT) to estimate focused attention at the behavioural level. The CPT was completed on a laptop computer. We adopted the A–X version of the CPT, in which a series of random one-digit numbers is displayed (total, 400 times), and the participant is instructed to press the spacebar as quickly as possible when the number “7” is immediately followed by the number “3.” The task lasted for 16 min 40 s, and the target stimuli occurred at a frequency of 10%. CPT performance was estimated based on reaction time (ms) and the coefficient of variation^53–56^. We used the number of commission errors, reaction time, and its coefficients of variation as indicators of attentional function.

### Acquisition of MRI data

**Figure 1:**
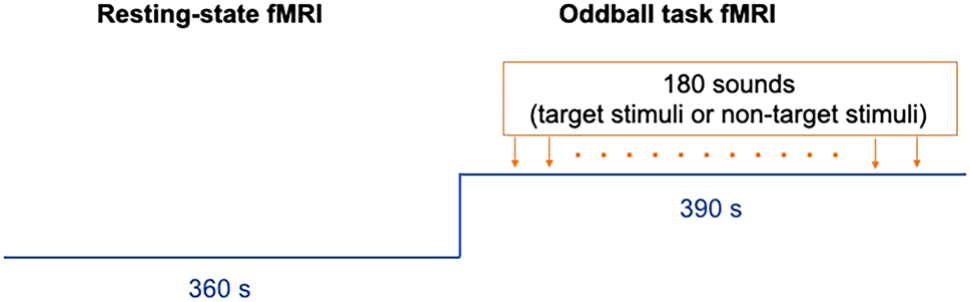
The method of MRI data acquisition. We analysed the entire task as a block as opposed to the Rest block. Abbreviation: fMRI = functional MRI.

As shown in Fig. 1, we used fMRI with an oddball (Odd) task—a basic attentional task with relatively low cognitive load—to minimize behavioural variance, as behavioural variance is considered a major shortcoming of fMRI studies^57^. MRI data were acquired using a 3-T MRI system (Tim-Trio; Siemens, Erlangen, Germany) with a 40 mT/m gradient and a receiver-only 32-channel phased-array head coil. An rs-fMRI scan was performed for 360 s, and the images were acquired using a single-shot gradient echo-planar imaging (EPI) pulse sequence. The participants were instructed to visually concentrate on a fixation cross in the centre of the screen and to avoid thinking about anything specific during resting-state (Rest) data acquisition. Next, they received a 25-s explanation of how to complete the auditory oddball task (Odd) and then performed the task for 390 s. The task consisted of 30 pink-noise sounds (target stimuli) and 150 pure 400-Hz tones (non-target stimuli)^58^ in a randomized order. All stimuli were presented for 200 ms with a jittered and randomized inter-stimulus interval of 1–3 s in 100-ms units. During the task, participants were instructed to differentiate between target and non-target stimuli by pressing a button with the right thumb as fast and accurately as possible after the target stimulus presentation. We measured the response time for all responses to the target stimuli. All participants practiced before entering the scanner, and we confirmed that they understood the procedure and were able to perform the 16-s practice sessions with 100% accuracy. The total fMRI acquisition time was 775 s. Head movement was minimized using foam rubber pads.

Structural MRI data were also acquired using three-dimensional magnetization-prepared rapid gradient-echo (3D-MPRAGE) sequences. The parameters for the 3D-MPRAGE images were as follows: echo time, 3.4 ms; repetition time, 2000 ms; inversion time, 990 ms; field of view, 225 × 240 mm; matrix size, 240 × 256; resolution, 0.9375  ×  0.9375  ×  1.0 mm^3^; and total axial sections without intersection gaps, 208. Parameters for the fMRI were as follows: echo time, 30 ms; repetition time, 2500 ms; flip angle, 80°; field of view, 212  ×  212 mm^2^; matrix size, 64 × 64; in-plane spatial resolution, 3.3125  ×  3.3125 mm^2^; total axial slices, 40; and slice thickness, 3.2 mm with 0.8 mm gaps in ascending order. A dual-echo gradient-echo dataset for B0-field mapping was also acquired for distortion correction.

### Image processing

We corrected the fMRI dataset for EPI distortion using field map data and FMRIB’s Utility for Geometrically Unwarping EPIs, which is part of the FSL package (FMRIB’s software library ver. 5.0.9; https://www.fmrib.ox.ac.uk/fsl), together with FMRIB's Linear Image Registration Tool (FLIRT) for realignment. We removed artifacts and motion-related fluctuations from the images using FMRIB’s ICA-based X-noisier (FIX). We then processed the preprocessed fMRI and structural MRI data using the CONN-fMRI Functional Connectivity toolbox (ver. 17f.; https://www.nitrc.org/projects/conn) with the statistical parametric mapping software package SPM12 (Wellcome Trust Centre for Neuroimaging; https://www.fil.ion.ucl.ac.uk/spm).

Before running FIX, we evaluated any movement that occurred during fMRI scanning using frame-wise displacement, which quantifies head motion between each volume of functional data. We applied two exclusion criteria: (1) when the number of volumes in which the head position was 0.5 mm different from those in adjacent volumes was more than 25% of the total volumes; and (2) when the maximum head motion was > 3.0 mm and > 3.0°. No participants were excluded under criterion 1 and three were excluded under criterion 2. Finally, 48 of 51 participants were included in the FC analysis.

All functional images were spatially normalised into the standard MNI space (Montreal Neurological Institute, Montreal, Canada), outliers were detected (ART-based scrubbing), and the images were smoothed using a Gaussian kernel with a full-width-at-half maximum of 8 mm using the customized CONN toolbox preprocessing pipeline. All preprocessing steps were conducted exclusively using a default preprocessing pipeline for volume-based analysis (to MNI space). Structural data were segmented into grey matter, white matter, and cerebrospinal fluid and normalised in the same default preprocessing pipeline. Principal components of signals from white matter and cerebrospinal fluid, as well as translational and rotational movement parameters (with another six parameters representing their first-order temporal derivatives) derived by realignment with FLIRT, were removed with covariate regression analysis using CONN. Using its implemented CompCor strategy32, the effects of nuisance covariates, including fluctuations in fMRI signals from white matter, cerebrospinal fluid, and their derivatives, as well as realignment parameter noise, were reduced. As recommended, band-pass filtering was performed with a frequency window of 0.008–0.09 Hz. This preprocessing step was found to increase retest reliability. We did not remove mean evoked responses prior to task-state FC analysis^59^.

### Analysis of functional connectivity

Regarding fMRI scans, 13 individuals were excluded from the study population because of the following reasons: seven gave no response to Odd during the paradigm; three were excluded based on criterion 1; and the remaining three were excluded based on criterion^2^. Finally, 48 of 61 participants were included in the FC analysis. We performed a region of interest (ROI)-to-ROI analysis using the CONN toolbox to examine the relationship between FCs and psychological indicators. The networks of interest were the DMN, FPN, and SN. We used ROIs from the widely used Stanford FIND atlas (https://greiciuslab.stanford.edu/resources^60–63^.

**Figure 2:**
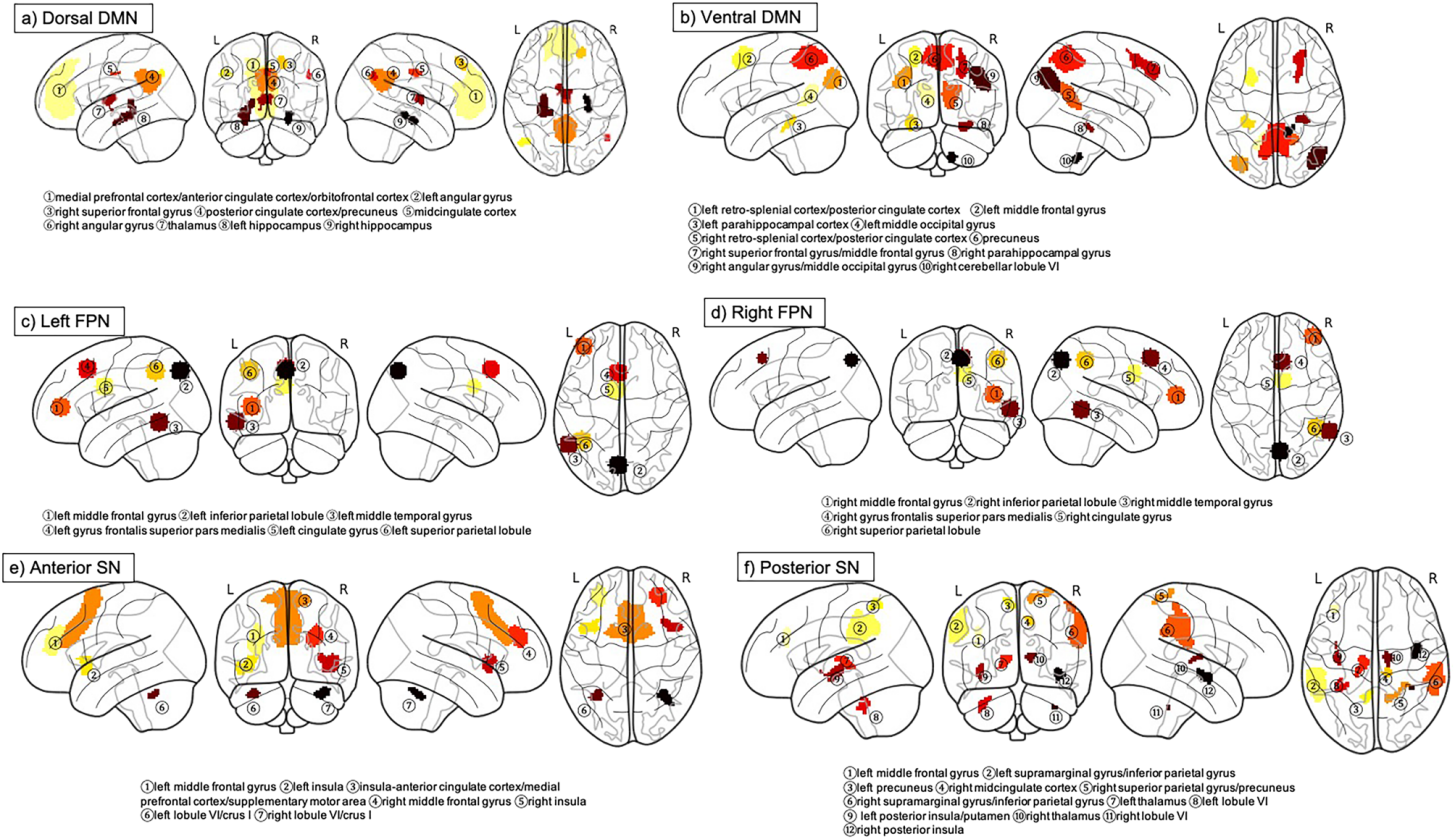
ROI locations according to the Stanford FIND atlas. The anatomical locations of 9 ROIs in the dorsal DMN (**a**), 10 ROIs in the ventral DMN (**b**), 6 ROIs in the left FPN (**c**), 6 ROIs in the right FPN (**d**), 7 ROIs in the anterior SN (**e**), and 12 ROIs in the posterior SN (**f**) are shown. The dorsal and ventral DMN, left and right FPN, and anterior and posterior SN are presented separately for improved visualization. Abbreviations: DMN = default mode network; FC = functional connectivity; FPN = frontoparietal network; ROI = region of interest; SN = salience network.

The nine ROIs in the dorsal DMN were located in the medial prefrontal cortex/anterior cingulate cortex/orbitofrontal cortex, thalamus, posterior cingulate cortex/precuneus, midcingulate cortex, right superior frontal gyrus, left and right angular gyrus, and left and right hippocampus^64,65^. The 10 ROIs in the ventral DMN were located in the left retro-splenial cortex/posterior cingulate cortex, left middle frontal gyrus, left parahippocampal cortex, left middle occipital gyrus, right retro-splenial cortex/posterior cingulate cortex, precuneus, right superior frontal gyrus/middle frontal gyrus, right parahippocampal gyrus, right angular gyrus/middle occipital gyrus, and right cerebellar lobule VI. The 12 ROIs in the FPN were bilaterally located in the middle frontal gyrus, inferior parietal lobule, middle temporal gyrus, gyrus frontalis superior pars medialis, cingulate gyrus, and superior parietal lobule. The seven ROIs in the anterior SN were located in the insula/anterior cingulate cortex/medial prefrontal cortex/supplementary motor area and bilaterally in the middle frontal gyrus, insula, and lobule VI/crus I. The 12 ROIs in the posterior SN were located in the left middle frontal gyrus, left supramarginal gyrus/inferior parietal gyrus, left precuneus, right midcingulate cortex, right superior parietal gyrus/precuneus, right supramarginal gyrus/inferior parietal gyrus, left and right thalamus, right lobule VI, left posterior insula/putamen, left lobule VI, and right posterior insula. Fig. 2 shows the anatomical locations of the 50 ROIs.

For each participant, we extracted and averaged the preprocessed fMRI time series of all voxels in the 50 ROIs. ROI-to-ROI FC was defined as the Fisher-transformed bivariate correlation coefficients for each pair of the regions. For second-level analysis, we used the subject-specific connectivity matrices for each ROI (as estimated from the CONN toolbox). We performed a one-way analysis of covariance with ASRS or AQ score as an independent variable, FC as a dependent variable, and age and sex as covariates of no interest. Significant connections were identified based on false discovery rate-corrected two-sided *p* values of < 0.05. False discovery rate corrections were made using CONN software, based on the default seed-level correction for 171 FC pairs in the analysis within the DMN, 66 within the FPN, 171 within the SN, 228 between the DMN and FPN, 361 between the DMN and SN, and 228 between the FPN and SN.

The ASRS and AQ scores may have interactive effects and influence overall FC. Therefore, we conducted separate analyses in which either the AQ or ASRS scores were independent variables and the others were considered covariates.

### Reproducibility assessment

To evaluate the reliability of the results of FC analyses, the 48 participants of the current study were randomly classified into two groups of 24 individuals, and each of the two groups was subjected to a ROI-to-ROI analysis using the CONN toolbox. Furthermore, the open-access datasets ADHD-200 (https://fcon_1000.projects.nitrc.org/indi/adhd200/) and ABIDE (https://fcon_1000.projects.nitrc.org/indi/abide/) were used to evaluate the reliability of our findings. In the ADHD-200 dataset, rs-fMRI data from healthy participants who completed the ADHD Rating Scale IV (ADHD-RS)^66^ were preprocessed and analysed using the CONN toolbox. Left-handed individuals were excluded, and the sample was restricted to those aged 12 years and older, resulting in a sample size of 47 participants (35 men, 13.4±0.8 years old). In the ABIDE, rs-fMRI data from healthy individuals who completed the Social Responsiveness Scale (SRS)^67^ were preprocessed and analysed using CONN. Left-handers were excluded, and the sample consisted of those aged 12 years and older. We used the ABIDE data acquired in a single site considering that the way of performing SRS (e.g., version, edition, and informant) as well as MRI data acquisition varied among each site, resulting in a sample size of 38 individuals (28 men, 14.6±1.6 years old).

Moreover, to evaluate the reproducibility of our findings, which were based on the FIND atlas, we additionally analysed our data using 15 ROIs from the default Harvard-Oxford Atlas (https://neuro.debian.net/pkgs/fsl-harvard-oxford-atlases.html) incorporated into the CONN Toolbox. The SN included 7 ROIs (anterior cingulate cortex, bilateral anterior insula, bilateral rostral prefrontal cortex, and bilateral supramarginal gyrus), the DMN 4 ROIs (medial prefrontal cortex, precuneus, and bilateral lateral parietal cortex), and the FPN 4 ROIs (bilateral lateral prefrontal cortex and bilateral posterior parietal cortex).

### Statistical analysis

The two-tailed *t*-test was used for between-group comparisons of demographic and behavioural data to examine how ASRS or AQ scores were associated with covariates such as CPT parameters, sex, and age. The one-sample Kolmogorov–Smirnov test revealed that the data showed mixed distributions. To test the above-mentioned correlations, we calculated Pearson’s correlation coefficients for normally distributed data and Spearman’s rank-correlation coefficients for non-normally distributed data. A *p* value < 0.05 was considered significant.

## Results

### Demographic characteristics

The demographic characteristics, ASRS and AQ scores, and CPT parameters of participants are summarized in Table 1. The mean AQ score was 21.9, which was almost identical to the value reported by a study that used the Japanese version of the AQ to assess a student population^49^. The mean ASRS score was 20.5, which was almost identical to the value reported by a study that used the Japanese version of the ASRS to assess members of the general population^46^.

**Table 1:** Demographic and behavioural data of the participants

Variables	Mean	Standard deviation	p*
Age (years)	21.9	1.9	0.014
ASRS total	20.5	10.1	0.200
ASRS inattention	13.5	6.1	0.090
ASRS hyperactivity	7.3	4.5	0.086
AQ total	21.9	7.6	0.079
AQ social skill	4.6	2.5	0.014
AQ attention switching	5.2	1.8	< 0.0001
AQ attention to detail	4.3	2.2	0.015
AQ communication	3.7	2.4	0.029
AQ imagination	2.2	2.2	0.001
CPT			
Commission error	1.7	1.5	0.200
Reaction time (ms)	412.1	60.0	< 0.0001
Standard deviation	78.5	38.3	0.012

*ASRS* Attention-Deficit Hyperactivity Disorder Self-Report Scale, *AQ* Autism-Spectrum Quotient, *CPT* continuous performance test.

*The *p* value in the Kolmogorov–Smirnov test.

### Psychological data

As shown in Table 2, we found a positive correlation between the ASRS and AQ scores (*r* = 0.447, *p* < 0.001). Among the ASRS subitems, both Inattention and Hyperactivity scores were positively correlated with AQ Total scores (ASRS Inattention: *r* = 0.342, *p* = 0.008; ASRS Hyperactivity: *r* = 0.440, *p* < 0.001). Many of the other subscales were also strongly correlated with each other.

**Table 2:** Correlation between psychological parameters.

		ASRS Total	ASRS Inattention	ASRS Hyperactivity	AQ Total	AQ Social Skill	AQ Attention Switching	AQ Attention to Detail	AQ Communication
ASRS inattention	*r*	0.901							
	*p*	< 0.001							
ASRS hyperactivity	*r*	0.868	0.664						
	*p*	0	0						
AQ total	*r*	0.447	0.44	0.342					
	*p*	< 0.001	< 0.001	0.008					
AQ social skill	*r*	0.279	0.365	0.157	0.757				
	*p*	0.033	0.004	0.234	< 0.001				
AQ attention switching	*r*	0.476	0.5	0.331	0.736	0.487			
	*p*	< 0.001	< 0.001	0.011	< 0.001	< 0.001			
AQ attention to detail	*r*	0.11	0.031	0.083	0.288	−0.138	0.06		
	*p*	0.409	0.814	0.533	0.027	0.297	0.651		
AQ communication	*r*	0.363	0.335	0.305	0.837	0.689	0.579	−0.04	
	*p*	0.005	0.01	0.019	< 0.001	< 0.001	< 0.001	0.766	
AQ imagination	*r*	0.318	0.284	0.307	0.757	0.437	0.454	0.131	0.554
	*p*	0.014	0.029	0.018	< 0.001	0.001	< 0.001	0.321	< 0.001

*ASRS* Attention-Deficit Hyperactivity Disorder Self-Report Scale, *AQ* Autism-Spectrum Quotient.

### Correlational between ADHD/ASD traits and CPT performance

The ASRS scores were negatively correlated with commission error, mean reaction time, and standard deviation on the CPT (Table 3). Among the ASRS subscales, the scores for Inattention were negatively correlated with commission error (*ρ* =  − 0.298, *p* = 0.019), whereas the scores for Hyperactivity were negatively correlated with mean reaction time (*ρ* =  − 0.258, *p* = 0.044). In contrast, AQ Total scores and all five subscale scores were not correlated with CPT performance.

**Table 3:** Correlations between ASRS/AQ scores and CPT performance with age and sex as covariates.

	Commission error		Mean reaction time (ms)		Standard deviation	
	*ρ*	*p*	*ρ*	*p*	*ρ*	*p*
ASRS total	−0.255	0.047	−0.275	0.032	−0.242	0.06
ASRS inattention	−0.298	0.019	−0.237	0.066	−0.159	0.222
ASRS hyperactivity	−0.195	0.012	−0.258	0.044	−0.157	0.228
AQ total	−0.041	0.755	−0.082	0.528	−0.121	0.352
AQ social skill	−0.173	0.182	0.012	0.925	−0.064	0.623
AQ attention switching	−0.146	0.261	−0.049	0.705	−0.141	0.277
AQ attention to detail	0.140	0.283	−0.157	0.226	−0.089	0.495
AQ communication	−0.018	0.888	−0.093	0.475	−0.080	0.538
AQ imagination	0.032	0.806	−0.127	0.330	−0.129	0.323

*ASRS* Attention-Deficit Hyperactivity Disorder Self-Report Scale, *AQ* Autism-Spectrum Quotient, *CPT* Continuous performance test.

### Correlations between ADHD/ASD scores and the FC of the SN, FPN, and DMN

**Figure 3:**
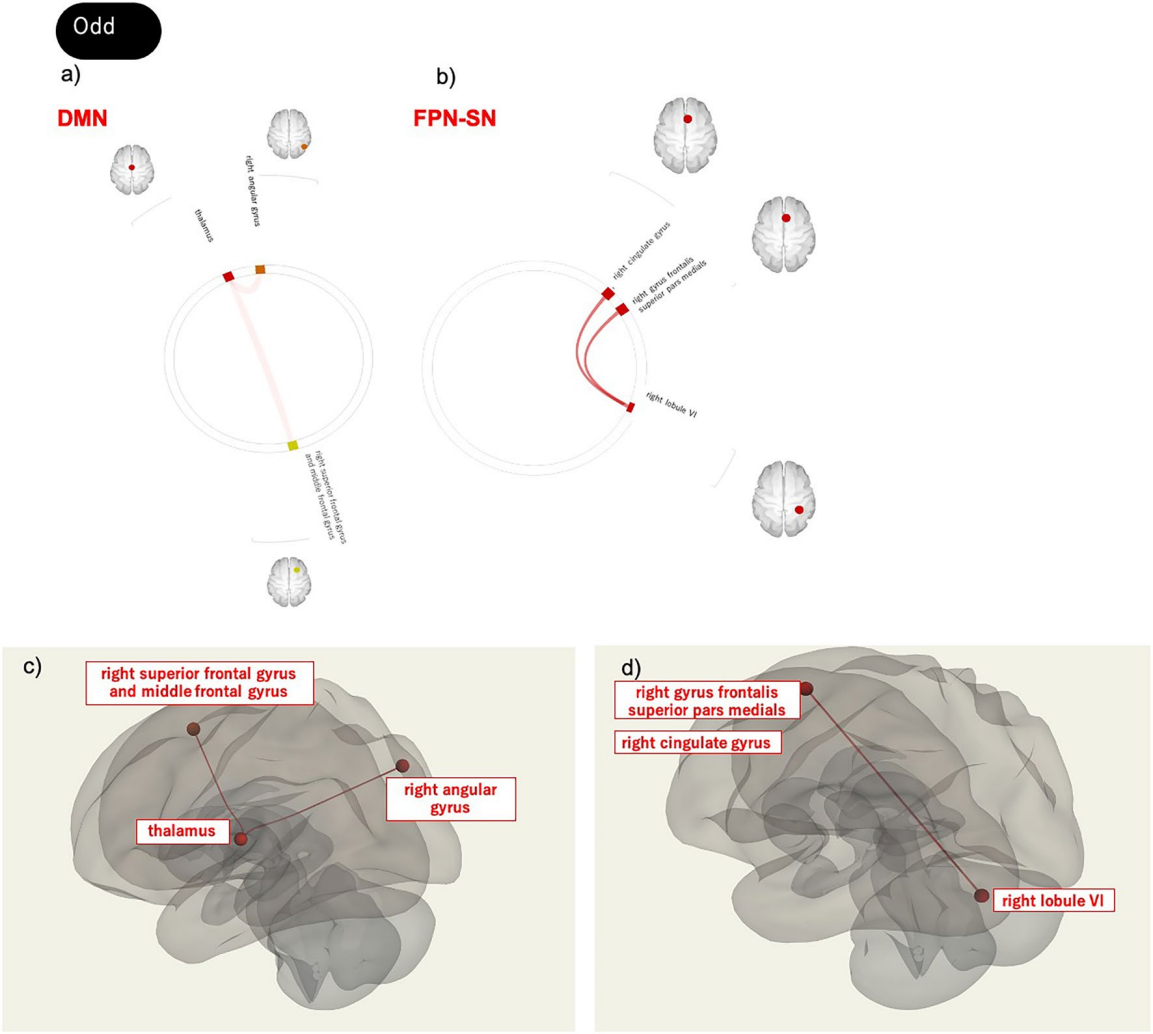
Network interactions that exhibit significant correlations between ASRS scores and FC values. (**a**, **c**) Network interactions that exhibit significant correlations between ASRS scores and FC values within the DMN during Odd. (**b**, **d**) Network interactions that exhibit significant correlations of ASRS scores with FC values between the FPN and SN during Odd. Abbreviations: ASRS = Attention-Deficit Hyperactivity Disorder Self-Report Scale; DMN = default mode network; FC = functional connectivity; FPN = frontoparietal network; Odd = oddball task; SN = salience network.

**Figure 4:**
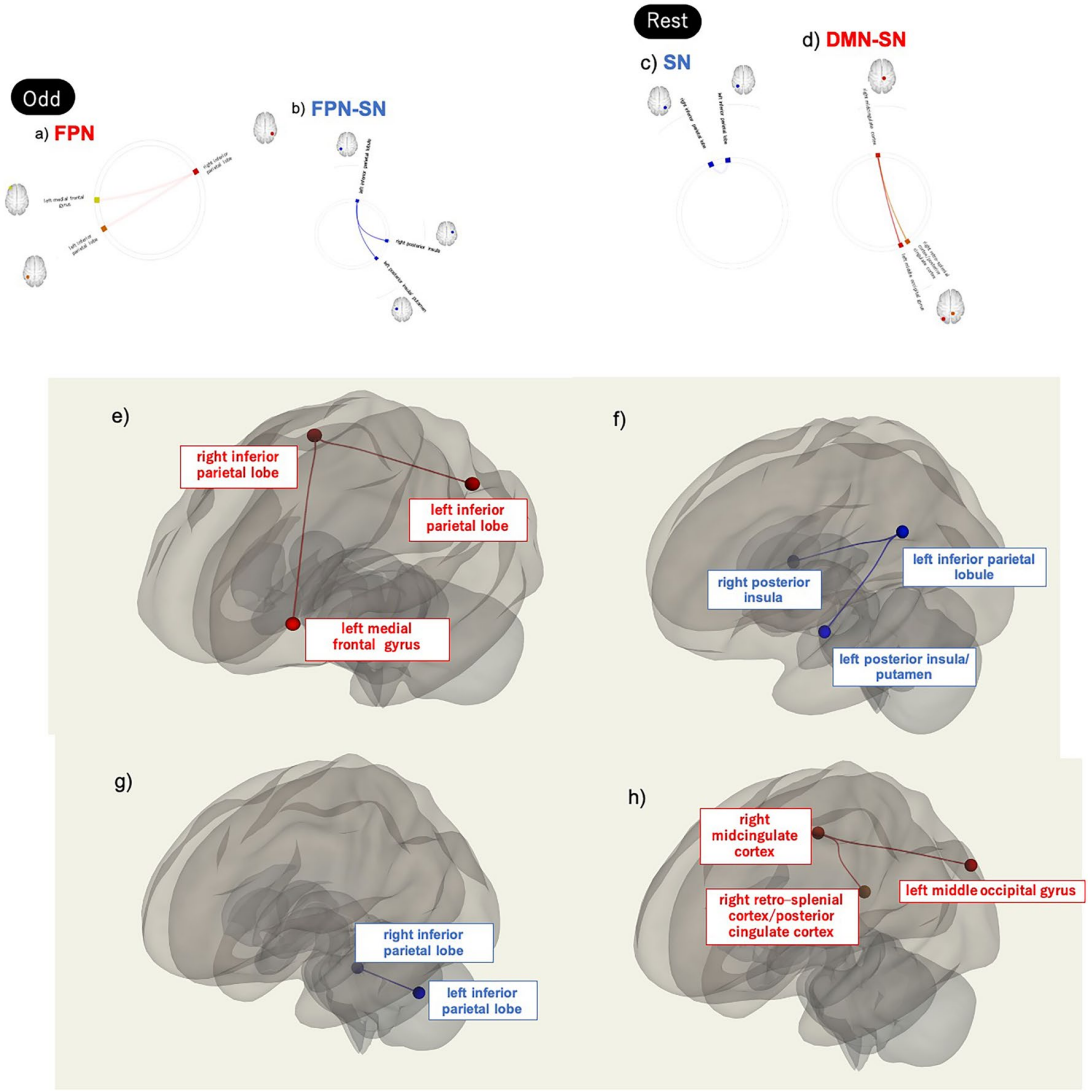
Network interactions that exhibit significant correlations between AQ scores and FC values. (**a**, **e**) Network interactions that exhibit significant correlations between AQ scores and FC values within the FPN during Odd. (**b**, **f**) Network interactions that exhibit significant correlations of AQ scores with FC values between the FPN and SN during Odd tasks. (**c**, **g**) Network interactions that exhibit significant correlations between AQ scores and FC values within the SN during Rest. (**d**, **h**) Network interactions that exhibit significant correlations of AQ scores with FC values between the DMN and SN during Rest. Abbreviations: AQ = Autism-Spectrum Quotient; DMN = default mode network; FC = functional connectivity; FPN = frontoparietal network; Odd = oddball task; Rest = resting state; SN = salience network.

During the Rest and Odd periods, the ASRS/AQ scores were differentially correlated with FC values between any two ROIs of the SN, FPN, and DMN (intra- or inter-network FC values; Tables 4, 5, 6, 7, 8, 9, 10, 11, 12 and Figs. 3, 4).

**Table 4:** Network interactions that exhibit significant correlations between ASRS scores and FC values.

		T-value (df = 44)	Uncorrected *p* value	FDR-adjusted *p* value	β
During odd					
Within the DMN	Right angular gyrus–thalamus	3.47	0.0012	0.00214	0.464
	Thalamus–right superior frontal gyrus and middle frontal gyrus	3.06	0.0037	0.0337	0.419
Between the FPN and SN	Right gyrus frontalis superior pars medialis–right lobule VI (CBM)	3.12	0.0032	0.0366	0.426
	Right cingulate gyrus–right lobule VI (CBM)	3.12	0.0032	0.0366	0.426

*ASRS* Attention-Deficit Hyperactivity Disorder Self-Report Scale, *FC* functional connectivity, *Odd* oddball task, *DMN* default mode network, *FPN* fronto-parietal network, *SN* salience network, *CBM* cerebellum, *df* degree of freedom, *FDR* false discovery rate, *β* standard partial regression coefficient.

**Table 5:** Network interactions that exhibit significant relationships between ASRS Inattention scores and FC values.

		T-value (df = 44)	Uncorrected *p* value	FDR-adjusted *p* value	β
During odd					
Between the FPN and SN	Right gyrus frontalis superior pars medialis–right lobule VI (CBM)	3.04	0.0040	0.0402	0.417
	Right cingulate gyrus–right lobule VI (CBM)	3.04	0.0040	0.0402	0.417
Between the DMN and SN	Left angular gyrus–right thalamus	−3.63	0.0007	0.0272	−0.480
During rest					
Within the FPN	Right middle frontal gyrus–right inferior parietal lobule	3.34	0.0017	0.0191	0.450
	Right middle frontal gyrus–left inferior parietal lobule	2.83	0.0069	0.0381	0.392
Within the DMN	Left middle occipital gyrus–posterior cingulate cortex/precuneus	−3.10	0.0034	0.0382	−0.423
	Right angular gyrus/middle occipital gyrus–left retro-Splenial cortex/posterior cingulate cortex	−3.06	0.0038	0.0374	−0.419
	Right angular gyrus/middle occipital gyrus–left parahippocampal cortex	−3.02	0.0042	0.0374	−0.045
	Left middle occipital gyrus–right angular gyrus	−2.99	0.0045	0.0382	−0.411
	Left middle occipital gyrus–right cerebellar lobule VI (CBM)	−2.87	0.0064	0.0382	−0.397
	Left middle occipital gyrus–left parahippocampal cortex	−2.59	0.0130	0.407	−0.364
	Left middle occipital gyrus–right hippocampus	−2.57	0.0135	0.407	−0.361
	Left middle occipital gyrus–right parahippocampal gyrus	−2.57	0.0136	0.407	−0.361

*ASRS* Attention-Deficit Hyperactivity Disorder Self-Report Scale, *FC* functional connectivity, Odd oddball task, *DMN* default mode network, *FPN* fronto-parietal network, *SN* salience network, *CBM* cerebellum, *df* degree of freedom, *FDR* false discovery rate, *β* standard partial regression coefficient.

**Table 6:** Network interactions that exhibit significant relationships between ASRS Hyperactivity scores and FC values.

		T-value (df = 44)	Uncorrected *p* value	FDR-adjusted *p* value	β
During odd					
Within the FPN	Right middle frontal gyrus–left cingulate gyrus	−3.10	0.0034	0.0369	−0.423
Within the DMN	Right angular gyrus–thalamus	3.34	0.0017	0.0308	0.450

*ASRS* Attention-Deficit Hyperactivity Disorder Self-Report Scale, *FC* functional connectivity, Odd oddball task, *FPN* fronto-parietal network, *DMN* default mode network, *df* degree of freedom, *FDR* false discovery rate, β standard partial regression coefficient.

**Table 7:** Network interactions that exhibit significant correlations between AQ scores and FC values.

		T-value (df = 44)	Uncorrected *p* value	FDR-adjusted *p* value	β
During odd					
Within the FPN	Left inferior parietal lobe–right inferior parietal lobe	3.41	0.0014	0.0155	0.457
	Left medial frontal gyrus–right inferior parietal lobe	2.91	0.0057	0.0314	0.402
Between the FPN and SN	Right inferior parietal lobule–right posterior insula	−3.16	0.0028	0.0423	−0.430
	Left middle occipital gyrus–left posterior insula/putamen	−3	0.0402	0.0439	−0.412
During rest					
Within the SN	Left lobule VI/crus I (CBM) –right lobule VI/crus I (CBM)	−3.3	0.0019	0.0351	−0.445
Between the DMN and SN	Left middle occipital gyrus–right midcingulate cortex	5	< 0.0001	0.0004	0.602
	Right retro–splenial cortex/posterior cingulate cortex–right midcingulate cortex	3.23	0.0023	0.0433	0.438

*AQ* Autism-Spectrum Quotient, *FC* functional connectivity, *Odd* oddball task, *DMN* default mode network, *FPN* frontal-parietal network, *SN* salience network, *CBM* cerebellum, *df* degree of freedom, *FDR* false discovery rate, *β* standard partial regression coefficient.

**Table 8:** Network interactions that exhibit significant correlations between AQ Social Skill scores and FC values.

		T-value (df = 44)	Uncorrected *p* value	FDR-adjusted *p* value	β
During odd					
Within the FPN	Left medial frontal gyrus–right inferior parietal lobe	3.27	0.0021	0.0231	0.442
Between the SN and FPN	Left posterior insula/putamen–left gyrus frontalis superior pars medialis	−3.24	0.0023	0.0374	−0.439
	Left posterior insula/putamen–right inferior parietal lobule	−3.08	0.0035	0.0374	−0.421
	Left posterior insula/putamen–left inferior parietal lobule	−3.06	0.037	0.0374	−0.419
	Left posterior insula/putamen–left inferior parietal lobule–right gyrus frontalis superior pars medialis	−2.78	0.0080	0.0419	−0.387
	Left posterior insula/putamen–right cingulate gyrus	−2.78	0.0080	0.0419	−0.387
	Left posterior insula/putamen–right middle frontal gyrus	−2.76	0.0084	0.0419	−0.384
During rest					
Between the SN and FPN	Right insula–right gyrus frontalis superior pars medialis	−3.64	0.0007	0.0108	−0.481
	Right insula–right cingulate gyrus	−3.64	0.0007	0.0108	−0.481
	Right insula–left gyrus frontalis superior pars medialis	−3.20	0.0025	0.0255	−0.435
	Right insula–left inferior parietal lobule	−2.90	0.0058	0.0438	−0.401
Between the SN and DMN	Right midcingulate cortex–left middle occipital gyrus	4.17	0.0001	0.0051	0.532
	Left middle frontal gyrus–right retro-splenial cortex/posterior cingulate cortex	3.29	0.0020	0.0380	0.444
	Left middle frontal gyrus–right cerebellar lobule VI (CBM)	3.28	0.0021	0.0380	0.443

*AQ* Autism-Spectrum Quotient, *FC* functional connectivity, *Odd* oddball task, *DMN* default mode network, *FPN* fronto-parietal network, *SN* salience network, *df* degree of freedom, *FDR* false discovery rate, *β* standard partial regression coefficient.

**Table 9:** Network interactions that exhibit significant relationships between AQ Attention Switching scores and FC values.

		T-value (df = 44)	Uncorrected *p* value	FDR-adjusted *p* value	β
During odd					
Between the SN and DMN	Right midcingulate cortex–left middle occipital gyrus	4.53	< 0.0001	0.0017	0.564
	Right thalamus–left angular gyrus	−3.77	0.0005	0.0179	−0.494
During rest					
Within the FPN	Right inferior parietal lobule–right middle frontal gyrus	3.76	0.0005	0.0055	0.493
Between the FPN and SN	Right inferior parietal lobule–left lobule VI/crus I (CBM)	3.58	0.0008	0.0127	0.475
Between the SN and DMN	Right midcingulate cortex–left middle occipital gyrus	5.34	< 0.0001	0.0001	0.627
	Right midcingulate cortex–right middle occipital gyrus	3.42	0.0013	0.0249	0.458
	Right midcingulate cortex–right retro-splenial cortex/posterior cingulate cortex	3.21	0.0025	0.0309	0.436

*AQ* Autism-Spectrum Quotient, *FC* functional connectivity, *Odd* oddball task, *DMN* default mode network, *FPN* fronto-parietal network, *SN* salience network, *df* degree of freedom, *FDR* false discovery rate, *β* standard partial regression coefficient.

**Table 10:** Network interactions that exhibit significant relationships between AQ Attention to Detail scores and FC values

		T-value (df = 44)	Uncorrected *p* value	FDR-adjusted *p* value	β
During odd					
Within the FPN	Right gyrus frontalis superior pars medialis–right middle temporal gyrus	−3.65	0.0007	0.0059	0.482
	Left gyrus frontalis superior pars medialis–right middle temporal gyrus	−3.50	0.0011	0.0059	0.467
Between the DMN and FPN	Thalamus–right middle temporal gyrus	3.12	0.0032	0.0319	0.426
During rest					
Between the SN and DMN	Left middle frontal gyrus–thalamus	3.77	0.0005	0.0177	0.494

*AQ* Autism-Spectrum Quotient, *FC* functional connectivity, *Odd* oddball task, *DMN* default mode network, *FPN* fronto-parietal network, *SN* salience network, *df* degree of freedom, *FDR* false discovery rate, *β* standard partial regression coefficient.

**Table 11:** Network interactions that exhibit significant relationships between AQ Communication scores and FC values.

		T-value (df = 44)	Uncorrected *p* value	FDR-adjusted *p* value	β
During odd					
Within the FPN	Right inferior parietal lobule–left middle frontal gyrus	3.10	0.0034	0.0265	0.423
	Right inferior parietal lobule–left inferior parietal lobule	2.97	0.0048	0.0265	0.409
During rest					
Within the SN	Right lobule VI/crus (CBM)–right posterior insula	−3.11	0.0033	0.0427	−0.425
	Right lobule VI/crus (CBM)–left supramarginal gyrus/inferior parietal gyrus	2.98	0.0047	0.0427	0.410
Between the FPN and DMN	Left middle temporal gyrus–midcingulate cortex	4.50	< 0.0001	0.0015	0.561
	Right inferior parietal lobule–right parahippocampal gyrus	3.37	0.0016	0.0471	0.453
Between the SN and DMN	Right midcingulate cortex–left middle occipital gyrus	3.55	0.0009	0.0283	0.472
	Right midcingulate cortex–right retro-splenial cortex/posterior cingulate cortex	3.38	0.0015	0.0283	0.454

*AQ* Autism-Spectrum Quotient, *FC* functional connectivity, *Odd* oddball task, *DMN* default mode network, *FPN* frontoparietal network, *SN* salience network, *df* degree of freedom, *FDR* false discovery rate, *β* standard partial regression coefficient.

**Table 12:** Network interactions that exhibit significant relationships between AQ Imagination scores and FC values.

		T-value (df = 44)	Uncorrected *p* value	FDR-adjusted *p* value	β
During odd					
Within the FPN	Right inferior parietal lobule–left inferior parietal lobule	3.25	0.0022	0.0246	0.440
	Right inferior parietal lobule–left middle frontal gyrus	2.83	0.0070	0.0387	0.392
Between the DMN and FPN	Medial prefrontal cortex/anterior cingulate cortex/orbitofrontal cortex–right middle temporal gyrus	−3.42	0.0013	0.0405	−0.458
	Medial prefrontal cortex/anterior cingulate cortex/orbitofrontal cortex–right gyrus frontalis superior pars medialis	−2.97	0.0049	0.0485	−0.409
	Medial prefrontal cortex/anterior cingulate cortex/orbitofrontal cortex–right cingulate gyrus	−2.97	0.0049	0.0485	−0.409
Between the SN and DMN	Left precuneus–right hippocampus	−3.58	0.0009	0.0319	−0.475
	Right lobule VI/crus (CBM)–left angular gyrus	−3.57	0.0009	0.0325	−0.474
During rest					
Within the SN	Left lobule VI (CBM)–right posterior insula	3.66	0.0007	0.0123	0.483
Within the DMN	Right hippocampus–right angular gyrus	3.59	0.0008	0.0151	0.476
Between the SN and DMN	Left middle frontal gyrus–left middle occipital gyrus	3.43	0.0013	0.0495	0.459

*AQ* Autism-Spectrum Quotient, *FC* functional connectivity, *Odd* oddball task, *DMN* default mode network, *FPN* frontoparietal network, *SN* salience network, *df* degree of freedom, *FDR* false discovery rate, *β* standard partial regression coefficient.

During Odd, total ASRS scores were positively correlated with multiple intra-network FC values within the DMN, such as those between the thalamus and right angular gyrus and those between the thalamus and right superior frontal gyrus and middle frontal gyrus. Total ASRS scores were also positively correlated with multiple inter-network FC values, such as those between the right gyrus frontalis superior pars medialis (FPN) and right cerebellar lobule VI (SN) as well as those between the right cingulate gyrus (FPN) and right cerebellar lobule VI (SN). During Rest, there were no correlations between total ASRS scores and intra- or inter-network FC values. During Odd, ASRS Inattention scores were positively correlated with multiple inter-network FC values between the FPN and SN and negatively correlated with multiple inter-network FC values between the DMN and SN. During Rest, ASRS Inattention scores were positively correlated with multiple intra-network FC values within the FPN and negatively correlated with multiple intra-network FC values between the FPN and DMN. During Odd, ASRS Hyperactivity scores were negatively correlated with one intra-network FC value within the FPN and positively correlated with one inter-network FC value within the DMN. During Rest, ASRS Hyperactivity scores were not correlated with intra- or inter-network FC values.

During Odd, the AQ scores were positively correlated with FC values between the left and right parietal lobes and those between the left frontal gyrus and right parietal lobe (FPN). We also found a negative correlation between AQ scores and two FC values: those between the right inferior parietal lobule (FPN) and right posterior insula (SN), and those between the left middle occipital gyrus (FPN) and left posterior insula/putamen (SN). During Rest, AQ scores were negatively correlated with intra-network FC values such as those between the left and right inferior parietal lobules (SN). However, the AQ scores were positively correlated with inter-network FC values such as those between the left occipital gyrus (DMN) and right midcingulate cortex (SN) and between the right retro-splenial cortex/posterior cingulate cortex (DMN) and right midcingulate cortex (SN). Regarding the correlation analysis with AQ subscales, during Odd, AQ Social Skill scores were positively correlated with multiple intra-network FC values within the FPN and negatively correlated with multiple inter-network FC values between the FPN and SN. During Rest, AQ Social Skill scores were positively correlated with multiple inter-network FC values between the SN and DMN and negatively correlated with multiple intra-network FC values within the FPN and SN. During Odd, AQ Attention Switching scores were positively or negatively correlated with multiple inter-network FC values between the DMN and SN. During Rest, AQ Attention Switching scores were positively correlated with multiple intra-network FC values within the FPN, as well as with multiple inter-network FC values between the FPN and SN and between the SN and DMN.

During Odd, AQ Attention to Detail scores were positively correlated with multiple inter-network FC values between the FPN and DMN and negatively correlated with one intra-network FC value within the FPN. During Rest, AQ Attention to Detail scores were positively correlated with one inter-network FC value between the SN and DMN. During Odd, AQ Communication scores were positively correlated with multiple intra-network FC values within the FPN. During Rest, AQ Communication scores were positively correlated with multiple inter-network FC values between the FPN and DMN and between the SN and DMN, and they were also negatively correlated with multiple intra-network FC values within the SN. During Odd, AQ Imagination scores were positively correlated with multiple intra-network FC values within the FPN and negatively correlated with multiple inter-network FC values between the DMN and FPN and between the SN and DMN. During Rest, AQ Imagination scores were positively correlated with multiple intra-network FC values within the SN and DMN and multiple inter-network FC values between the FPN and SN and between the SN and DMN.

**Figure 5:**
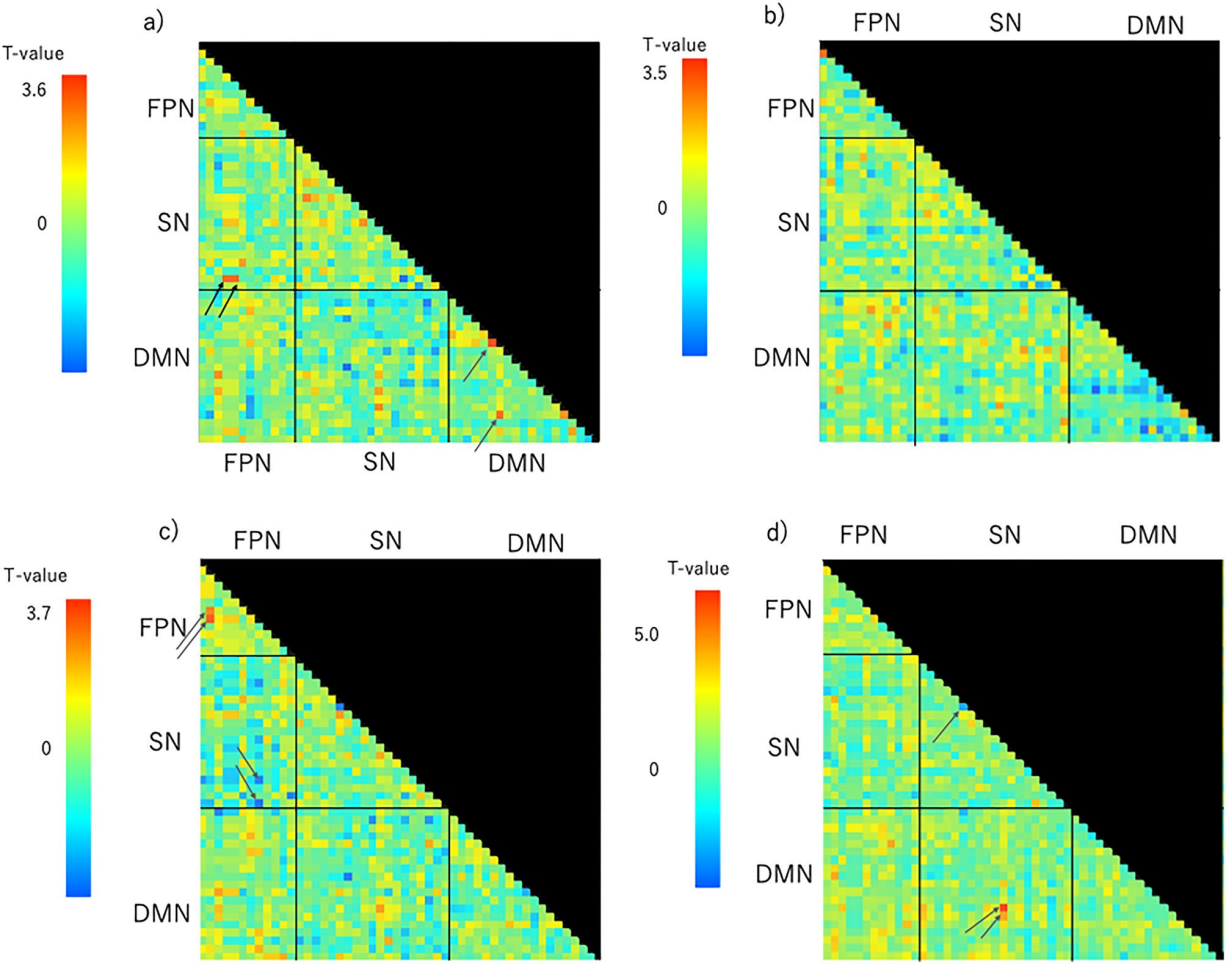
T-value matrix plots of the network interactions between ASRS or AQ scores and FC values. Connectivity matrix for ASRS Total scores as an independent variable during tasks (**a**) and resting state (**b**). Connectivity matrix for AQ Total scores as an independent variable during tasks (**c**) and resting state (**d**). Cells in yellow-red indicate a positive pairwise correlation between two regions of interest; green-blue cells indicate a negative pairwise correlation. The anatomical labels for each intrinsic connectivity network are from top to bottom on the x-axis and from left to right on the y-axis as follows: right middle frontal gyrus (FPN), right inferior parietal lobule (FPN), right middle temporal gyrus (FPN), right gyrus frontalis superior pars medialis (FPN), right cingulate gyrus (FPN), right superior parietal lobule (FPN), left middle frontal gyrus (FPN), left inferior parietal lobule (FPN), left middle temporal gyrus (FPN), left gyrus frontalis superior pars medialis (FPN), left cingulate gyrus (FPN), left superior parietal lobule (FPN), left middle frontal gyrus (anterior SN), left insula (anterior SN), insula-anterior cingulate cortex/medial prefrontal cortex/supplementary motor area (anterior SN), right middle frontal gyrus (anterior SN), right insula (anterior SN), left lobule VI/crus I (anterior SN), right lobule VI/crus I (anterior SN), left middle frontal gyrus (posterior SN), left supramarginal gyrus/inferior parietal gyrus (posterior SN), left precuneus (posterior SN), right midcingulate cortex (posterior SN), right superior parietal gyrus/precuneus (posterior SN), right supramarginal gyrus/inferior parietal gyrus (posterior SN), left thalamus, left lobule VI (posterior SN), left posterior insula/putamen (posterior SN), right thalamus, right lobule VI (posterior SN), right posterior insula (posterior SN), medial prefrontal cortex/anterior cingulate cortex/orbitofrontal cortex (dorsal DMN), left angular gyrus (dorsal DMN), right superior frontal gyrus (dorsal DMN), posterior cingulate cortex/precuneus (dorsal DMN), midcingulate cortex (dorsal DMN), right angular gyrus (dorsal DMN), thalamus (dorsal DMN), left hippocampus (dorsal DMN), right hippocampus (dorsal DMN) , left retro-splenial cortex/posterior cingulate cortex (ventral DMN), left middle frontal gyrus (ventral DMN), left parahippocampal cortex (ventral DMN), left middle occipital gyrus (ventral DMN), right retro-splenial cortex/posterior cingulate cortex (ventral DMN), precuneus (ventral DMN), right superior frontal gyrus/middle frontal gyrus (ventral DMN), right parahippocampal gyrus (ventral DMN), right angular gyrus/middle occipital gyrus (ventral DMN), and right cerebellar lobule VI (ventral DMN). Abbreviations: AQ = Autism-Spectrum Quotient; ASRS = Attention-Deficit Hyperactivity Disorder Self-Report Scale; DMN = default mode network; FC = functional connectivity; FPN = frontoparietal network; SN = salience network.

Fig. 5 shows the T-value matrix plots of the network interactions between ASRS or AQ scores and FC values of the 50 ROIs in the FPN, SN, and DMN when either AQ or ASRS Total scores were used as an independent variable. The ROI connectivity matrices were separately generated for Rest and Odd. These matrix plots largely confirm the above results of the network interactions between ASRS or AQ scores and FC values. These plots show especially many negative correlations between ASRS Total scores and FC values within the DMN during Rest and between AQ Total scores and FC values between the SN and FPN during Odd.

The results of the relationships of ASRS and AQ Total scores with FC values were similar to those obtained when either AQ or ASRS was used as an independent variable and the other was used as a covariate (see Supplementary Tables S1 and S2, Additional File 1). We also performed FC analyses focusing on ASRS and AQ subscale scores. The psychological parameter–FC relationships differed between the two conditions (i.e. the condition that each subscale was treated as an independent variable alone [without covariates] and the condition that other subscales were included as covariates in the statistical models) (see Supplementary Tables S3–S9, Additional File 1).

Moreover, when both AQ and ASRS were used as independent variables, during Odd, AQ and ASRS Total scores were positively correlated with FC values between the left inferior parietal lobule and left middle temporal gyrus within the FPN. During Rest, those scores were also positively correlated with FC values between the midcingulate cortex (DMN) and left middle temporal gyrus (FPN) and between the left middle occipital gyrus (DMN) and right midcingulate cortex (SN; see Supplementary Table S10, Additional File 1).

### Reproducibility assessment

When the 48 participants were randomly divided into Groups 1 and 2, the results of the split-half analysis did not fully replicate the results from the entire study population but were partially consistent with the above results (see Supplementary Tables S11–S14, Additional File 1). Similar to our findings in the entire population, total ASRS scores were positively correlated with one intra-network FC value within the DMN during Odd within Group 2. The results that differed from our findings in the entire study population include the following. (1) Within Group 1, Total ASRS scores were positively correlated with multiple intra-network FC values within the SN and negatively correlated with one inter-network FC value between the DMN and SN during Odd and between the DMN and FPN during Rest. (2) Within Group 2, total ASRS scores were positively correlated with one intra-network FC value within the DMN during Rest. (3) AQ scores within Group 1 were positively and negatively correlated with intra-network FC values within the SN, negatively correlated with one inter-network FC value between the DMN and SN during Odd, and positively correlated with one FC values between the DMN and FPN during Rest. (4) Within Group 2, AQ scores were positively correlated with one intra-network FC value within the DMN during both Odd and Rest.

As for the reproducibility assessment using open-access datasets, the analysis of the ADHD-200 dataset did not replicate the results of the current study (see Supplementary Tables S15, S16, Additional File 1). During Rest, ADHD-RS scores were positively correlated with intra-network FC values within the SN and with inter-network FC values between the DMN and FPN. ADHD-RS Inattention scores were not correlated with intra- or inter-network FC values. During Rest, ADHD-RS Hyperactivity scores were positively correlated with intra-network FC values within the SN and within the FPN.

The analysis of the ABIDE dataset also did not fully replicate the results of this study but was partially consistent with our results using AQ Total, Social skills, Attention switching, Attention to Detail, Imagination and Communication scores (see Supplementary Table S17, Additional File 1). Similar to our study findings, the SRS scores were positively correlated with multiple inter-network FC values between the DMN and SN during Rest. The results that differ from our findings include the following: During Rest, the SRS scores were negatively correlated with multiple intra-network FC values within the FPN and within the DMN, negatively correlated with one inter-network FC value between the DMN and SN, and positively correlated with one inter-network FC value between the DMN and FPN.

When we additionally analysed the data using ROIs from the default Harvard-Oxford Atlas, the results of the analysis using ROIs from the Stanford FIND atlas were not completely replicated but were partially consistent (see Supplementary Tables S18, S19, Additional File 1). During Odd, ASRS scores were not correlated with intra- or inter-network FC values. During Rest, ASRS scores were negatively correlated with one intra-network FC value within the DMN. During Odd, the AQ scores were negatively correlated with one inter-network FC value between the FPN and SN, positively correlated with multiple intra-network FC values within the FPN, and positively correlated with one inter-network FC value between the DMN and FPN. During Rest, the AQ scores were positively correlated with one intra-network FC value within the FPN.

## Discussion

**Figure 6:**
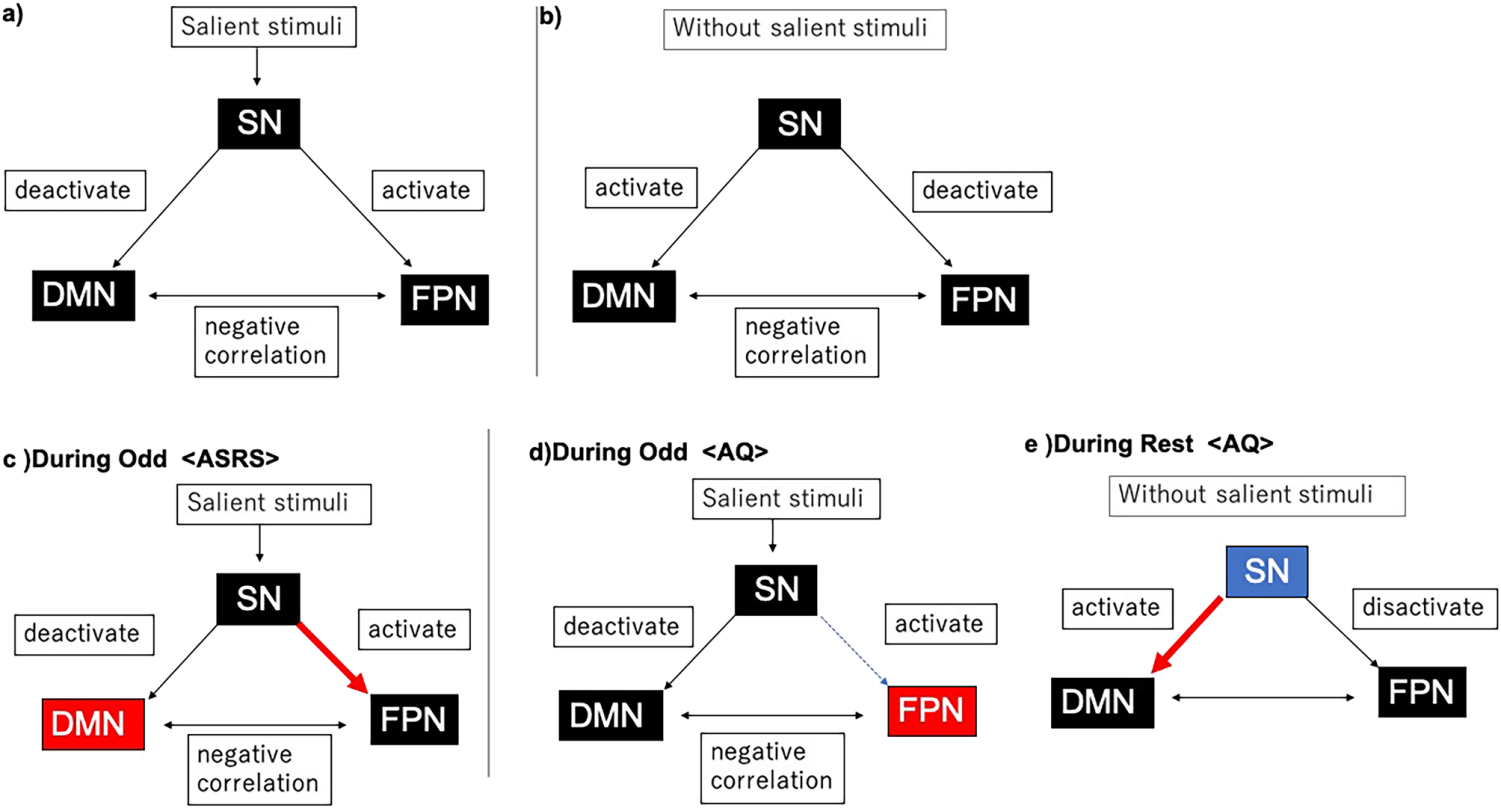
Relationships between ASRS or AQ scores and the SN, FPN, and DMN. (**a**) The SN activates the FPN and deactivates the DMN in response to salient stimuli, and (**b**) it activates the DMN and deactivates the FPN during Rest. (**c**) During Odd, higher ASRS scores are correlated with increased FC values within the DMN and increased FC values between the FPN and SN, whereas (**d**) higher AQ scores are correlated with increased FC values within the FPN and decreased FC values between the FPN and SN. (**e**) During Rest, only high AQ scores are correlated with decreased FC values within the SN and increased FC values between the DMN and SN. Red arrows reflect increased FC values between network pairs, whereas blue dotted arrows reflect decreased FC values. Red boxes reflect increased FC values within network pairs, whereas blue boxes reflect decreased FC values. Abbreviations: AQ = Autism-Spectrum Quotient; ASRS = Attention-Deficit Hyperactivity Disorder Self-Report Scale; DMN = default mode network; FC = functional connectivity; FPN = frontoparietal network; Odd = oddball task; Rest = resting condition; SN = salience network.

In this study, we examined the correlation between ADHD/ASD traits and the intra-/inter-network integrity of the SN, FPN, and DMN in subclinical populations. At the behavioural level, ASRS Total, Inattention, and Hyperactivity scores (i.e. ADHD traits) were positively correlated with performance in the CPT, whereas AQ Total and all subitem scores (i.e. ASD traits) were not. In the FC analyses, the ASRS and AQ scores were differentially correlated with intra- and inter-network connectivity among various ROIs in the DMN, SN, and FPN. Using either the FIND or Harvard-Oxford atlas, during Odd, ASRS scores were positively correlated with FC values within the DMN, whereas AQ scores were positively correlated with those within the FPN. The AQ scores were negatively correlated with FC values between the FPN and SN. Using the FIND atlas, during Odd, the ASRS scores were positively correlated with FC values between the FPN and SN. During Rest, only AQ scores were negatively correlated with one FC value within the SN, and AQ scores were positively correlated with multiple FC values between the DMN and SN. Other than the negative correlation of AQ with one FC value within the SN during Rest, these findings of the ROI-to-ROI analysis were not replicated in the split-half replication analysis or in the replication analysis using open-access datasets. Fig. 6 shows the relationships of AQ and ASRS scores with the SN, FPN, and DMN. The differences in ASRS/AQ–FC correlations may be explained by the fact that the subscales are not statistically orthogonal.

### Relationship between ADHD/ASD traits and CPT performance

Adults and children with ADHD generally show lower performance on the CPT^68–70^. However, there are no consistent reports regarding the association between ASD and CPT performance^70,71^.

Our results indicate that the higher the ASRS score, the more favourable the CPT performance and the shorter the reaction time. This suggests that ADHD traits beneath the diagnostic threshold may promote attentional retention functions. A previous questionnaire study reported that CPT performance was intact in healthy children with low-to-moderate ADHD symptoms, indicating that CPT performance can vary widely among different levels of ADHD severity^72^.

To the best of our knowledge, this study is the first to indicate an association between favourable CPT performance and susceptibility to subclinical-level ADHD. Moderate levels of media multitasking are expected to be associated with enhanced cognitive control, and several reports suggest an inverted U-shaped relationship between attentional performance and media multitasking^73,74^. Adults with ADHD are known to choose multitasking tasks quite often^75^. Considering the potential preference for multitasking in individuals with subclinical ADHD, intermediate-level ADHD traits are also expected to demonstrate an inverted U-shaped relationship with attentional processing. If the inverted U-shaped model is accepted, the participants in the present study would be distributed on the left side of the curve. In particular, those with higher (but still subclinical) ADHD traits would be located near the apex.

Two hypotheses have been proposed regarding the relationship between media multitasking and attentional functioning: the scattered attention hypothesis and the trained attention hypothesis. The scattered attention hypothesis argues that long-term media multitasking weakens attentional control, meaning that individuals exposed to a multitasking lifestyle are less capable of maintaining focus on relevant tasks in the presence of distractions^73^. In contrast, the trained attention hypothesis argues that frequent multitasking enhances cognitive control and positively affects attention^76^. The results of the current study suggest that individuals with subclinical ADHD perform better in the CPT, which may be consistent with the trained attention hypothesis.

### Relationship between ADHD traits and the SN, FPN, and DMN

Previous studies reported functional deficits in the DMN among individuals with ADHD^28,29,76–82^. The DMN is commonly activated when the brain is free from external cognitive demands or when it is performing internal tasks^83,84^. However, individuals with ADHD exhibit reduced FCs in the DMN during the resting state and reduced inhibitory changes in FCs during task performance^40,85,86^. Our results also showed that ASRS Total and Hyperactivity scores were positively correlated with the intra-network integrity of the DMN during tasks and that ASRS Inattention scores were negatively correlated with the intra-network integrity of the DMN during Rest. These results indicated that, in terms of DMN activities, people with subclinical ADHD traits and those with clinical-level ADHD share some common neural bases.

Our analysis of inter-network relationships among the SN, FPN, and DMN showed that ASRS Total and Inattention scores of subclinical individuals were positively associated with the integrity of SN–FPN FCs. To the best of our knowledge, no other study has reported increased FC between the SN and FPN during tasks in individuals with ADHD. Some earlier studies in children with clinical-level ADHD showed reduced FC between parietal and cerebellar brain regions during cognitive tasks involving attention and response inhibition^87,88^. Previous studies in the general population have suggested that the FC between the SN and FPN—including the FC between the cerebellum (involved in the SN) and prefrontal cortex (involved in the FPN)—increases in response to cognitive demands, especially during auditory cognitive tasks^89–92^. In the current study, the SN ROI related to increased FC was limited to cerebellar lobule VI. Previous studies also have shown that the posterior cerebellar lobes, including lobule VI, are activated during cognitive tasks requiring working memory and executive function^92,93^. Thus, the positive associations between ADHD traits and the SN (cerebellum)–FPN (frontal gyrus, including the cingulate) FCs during the oddball task (as observed in the current study) may indicate improved sustained attentional functions (insofar as the level of ADHD traits is lower than intermediate). The increased SN–FPN FCs may also explain the results of the behavioural part of this study—that is, improved performance in the CPT in individuals with a higher (but still subclinical) level of ADHD traits.

### Relationship between ASD traits and the SN, FPN, and DMN

Our analysis of intra- and inter-network FCs revealed differential changes in FCs in association with ASD traits. Our results are partially consistent with those of previous rs- and task-based fMRI studies^25,29,41,94–97^. In particular, we noted decreased FCs between the SN and FPN during tasks associated with AQ Total and Social Skill scores, decreased FCs between the SN and DMN during tasks associated with AQ Imagination scores, decreased FCs within the SN during Rest associated with AQ Total and Communication scores, increased FCs between the SN and DMN during Rest associated with AQ Total and all five subscale scores, and decreased FCs between the SN and FPN during Rest associated with AQ Social Skill scores.

The SN detects salient internal and external stimuli and regulates other brain regions by switching the brain networks^8,36^. Therefore, the decrease in FCs within the SN and their associations with symptom severity^94,98^ suggest that the SN may contribute to both overactive and underactive brain functions in individuals with ASD. For example, emotion dysregulation—a common clinical symptom of ASD^99,100^—may be explained by the failure of the cognitive control of emotion (i.e. the FPN function) caused by problems in attention when “selecting” stimulus importance (i.e. the “switching” function of the SN). Previous studies have hypothesized that increased SN–DMN FC is potentially associated with repetitive negative self-thoughts in ASD^38^. Previous fMRI studies have also reported that, in depression, dysfunction within the SN contributes to abnormal engagement and disengagement of the DMN and FPN. This may cause difficulties in disengaging the processing of negative information, thus contributing to the worsening of negative thoughts^101,102^. The mechanism behind negative thoughts in ASD may be similar to that in depression, which is often comorbid with ASD^103,104^.

ASD traits were negatively associated with FCs within the SN (during Rest) and those between the SN and FPN (during Odd). This indicated that, in terms of selective attentional processing, even people with subclinical ASD traits share common neural bases with those with clinical-level ASD. This may cause problems in attention when “selecting” stimulus importance. ASD traits were positively associated with the FCs of the FPN during Odd; this is consistent with the hypothesis that, in ASD adults, sustained attention is not significantly different from that in people with typical development^105,106^.

### Potential common neural bases of ADHD and ASD traits

When both AQ and ASRS were used as independent variables, during Odd, AQ and ASRS total scores were positively correlated with FC values within the FPN. During Rest, those scores were positively correlated with FC values between the DMN and FPN and between the DMN and SN. A prior study showed that hyperconnectivity between the DMN and FPN during Rest was found in a group with comorbid clinical-level ASD and ADHD^107^. This indicates that people with comorbid ADHD and ASD at the subclinical level and those at the clinical level share some common neural bases.

Regarding ASRS and AQ subscales, both AQ Attention Switching and ASRS Inattention scores were negatively correlated with FC values between the right thalamus (SN) and left angular gyrus (DMN) during Odd and were positively correlated with FC values of the right middle frontal gyrus and right inferior parietal lobule within the FPN during Rest. Both AQ Attention to Detail and ASRS Hyperactivity were negatively correlated with FC values within the FPN during Odd. Both AQ Imagination and ASRS Inattention scores were negatively correlated with FC values between the DMN and SN during Odd. Among the several similar network interactions shared among the subscales, the similarity in the network interactions between AQ Attention Switching and ASRS Inattention is remarkable. The correlation between these two subitems was also found in previous studies^108,109^. This suggests that these traits share similar neural bases. However, different meanings of ASRS Inattention and AQ Switching subscales have been suggested previously^110,111^, Therefore, the commonality in neural correlates of these subscales should be cautiously interpreted.

### Reproducibility assessment

When the 48 participants were randomly divided into two groups for a split-half analysis, the results were partially consistent with the initial findings, although they did not fully replicate those of the entire study population, possibly because the two subgroups were, with 24 participants each, quite small.

The analysis of the ADHD-200 dataset did not replicate the results of the current study. However, the results of the ABIDE data analyses were consistent with our results, especially regarding AQ Total and AQ Social Skills, Attention Switching, Attention to Detail, Imagination, and Communication scores, but only partially. The following factors may have contributed to the inability to reproduce our study results: the participants in the ADHD-200 and ABIDE data were younger than those in our study, they were not Japanese and (as in our study), the participants were predominantly male, the rating scales were different (ADHD-RS instead of ASRS, SRS instead of AQ), and the evaluation was limited to Rest.

When we additionally analysed our data using ROIs from the Harvard-Oxford Atlas, the results of the analysis were not completely replicated but were partially consistent. Whether using the Stanford FIND atlas or the Harvard-Oxford Atlas, during Odd, the AQ scores were negatively correlated with inter-network FC values between the FPN and SN and positively correlated with intra-network FC values within the FPN. The network interactions that could not be reproduced may be related to the fact that the Harvard-Oxford atlas did not include the cerebellum and other regions that are included in the FIND atlas. Specifically, the positive associations between ADHD traits and the SN–FPN FCs during Odd are expected to not be replicated, as they involved the cerebellum. In this study, the inclusion of the cerebellum in the atlas is worthwhile in light of the following facts. Anatomical tracing, human clinical, and stimulation functional imaging studies have firmly established the major role of the cerebellum in cognition and emotion processing^92,112–115^. Previous studies have also demonstrated that the cerebellum participates in many intrinsic connective networks, including the SN and DMN^116,117^. Moreover, cerebellar abnormalities in ASD and ADHD have been confirmed by functional and structural MRI; disruptions in cerebellar function may lead to social and cognitive deficits in ASD^118–120^, while cerebellar dysfunction in ADHD may lead to time perception deficits, emotional deficits, and childhood motor deficits^121–125^. Another possible limitation of the replications may be that the Harvard-Oxford Atlas is a structural atlas, which does not properly represent a functional analysis when compared with the FIND atlas^63,126^.

### Limitations

Our study has some limitations. First, the sample size was relatively small, and the number of individuals with clinical ASD or ADHD traits was quite small. A total of nine participants were screened as positive for ADHD according to Part A of the ASRS. Using a cut-off score of 33 for the AQ^127^, six participants were screened as positive for ASD. Finally, four participants who had been screened as positive for ADHD exceeded the cut-off score for the AQ. Second, another major limitation is the lack of reproducibility of our findings both within our study population (split-half replication) and in out-of-sample replication (ADHD-200 and ABIDE datasets). Third, we did not examine continuity in attentional function and neural substrates among participants with clinical-level traits. Because we recruited only participants with subclinical traits, we could only evaluate continuity based on comparisons with the results of prior studies. Further studies are needed to perform direct comparisons between patients with subclinical traits versus those with clinical traits. Finally, the present study used the CPT to assess attentional function. However, this test is known to be less sensitive in adolescents with ADHD than in children with ADHD. Moreover, there was no significant difference in performance between high-functioning individuals with ADHD and people with typical development^54,128^. Reports suggested that the task difficulty of CPT may be too low to measure attentional function. Some studies have suggested that adding complex distractor stimuli to the CPT can increase the differences in performance between people with ADHD and typically developing individuals. In the present study, we considered the possibility that ADHD traits may facilitate the maintenance of attention. However, future studies should compare performances between individuals with ADHD and typically developing controls using more complex attentional tasks.

## Conclusions

Our fMRI findings revealed that ASRS and AQ scores were differentially associated with the FCs of different LSBNs, indicating differential mechanisms of attentional processing in individuals with subclinical ADHD or ASD traits. This study indicates that brain imaging may assist in differentiating ADHD and ASD traits when assessing inattentive symptoms associated with these disorders.

### Supplementary Information

Supplementary Tables.

## Data Availability

The datasets used and/or analysed during the current study are available from the corresponding author on reasonable request.

## Abbreviations

ADHD: Attention-deficit hyperactivity disorder
AQ: Autism spectrum quotient
AS: Asperger’s syndrome
ASD: Autism spectrum disorder
ASRS: Adult ADHD self-report scale
CPT: Continuous performance test
DMN: Default mode network
EPI: Echo-planar imaging
FC: Functional connectivity
FIX: FMRIB’s ICA-based X-noisier
FLIRT: FMRIB's linear image registration tool
fMRI: Functional MRI
FPN: Fronto-parietal network
HFA: High-functioning autism
LSBN: Large-scale brain network
MPRAGE: Magnetization-prepared rapid gradient-echo
ROI: Region of interest
rs-fMRI: Resting-state fMRI
SN: Salience network
