# Supplementary material for: Differences between subclinical attention-deficit/hyperactivity and autistic traits in default mode, salience, and frontoparietal network connectivities in young adult Japanese
Source: Sci Rep. 2023 Nov 13;13:19724. doi: 10.1038/s41598-023-47034-7 (PMC10643712; doi:10.1038/s41598-023-47034-7)
Supplement: Supplementary file 1 — Supplementary Tables. [file 41598_2023_47034_MOESM1_ESM.docx]

**Supplementary Information**

**Differences between subclinical attention-deficit/hyperactivity and autistic traits in default mode, salience, and frontoparietal network connectivities in young adult Japanese**

Risa Hirata, Sayaka Yoshimura, Key Kobayashi, Morio Aki, Mami Shibata, Tsukasa Ueno, Takashi Miyagi, Naoya Oishi, Toshiya Murai & Hironobu Fujiwara

**Supplementary Table S1.** Network interactions that exhibit significant relationships between ASRS scores and FC values when AQ score, age, and sex were used as covariates of no interest.

|  |  | **T-value**  **(df = 43)** | **Uncorrected *p*-value** | **FDR-adjusted *p*-value** | **β** |
| --- | --- | --- | --- | --- | --- |
| During Odd |  |  |  |  |  |
| Within the DMN | right angular gyrus–thalamus | 3.53 | 0.0010 | 0.00178 | 0.474 |
|  | thalamus–right superior frontal gyrus and middle frontal gyrus | 3.04 | 0.0041 | 0.0366 | 0.421 |
| Between the FPN and DMN | left middle frontal gyrus–right superior frontal gyrus/middle frontal gyrus | -3.37 | 0.008 | 0.0478 | -0.457 |

Abbreviations: ASRS = Attention-Deficit Hyperactivity Disorder Self-Report Scale; FC = functional connectivity; AQ = Autism Spectrum Quotient; Odd= oddball task; DMN = default mode network; FPN = fronto-parietal network; df = degree of freedom; FDR = false discovery rate; β = standard partial regression coefficient

**Supplementary Table S2.** Network interactions that exhibit significant relationships between AQ scores and FC values when ASRS score, age, and sex were used as covariates of no interest.

|  |  | **T-value**  **(df = 43)** | **Uncorrected *p*-value** | **FDR-adjusted *p*-value** | **β** |
| --- | --- | --- | --- | --- | --- |
| During Odd |  |  |  |  |  |
| Between the FPN and SN | right inferior parietal lobule–right posterior insula | -3.72 | 0.0006 | 0.0173 | -0.493 |
|  | left middle occipital gyrus–left posterior insula/ putamen | -3.00 | 0.0022 | 0.0334 | -0.416 |
| During Rest |  |  |  |  |  |
| Within the SN | left inferior parietal lobe–right inferior parietal lobe | -3.85 | 0.0033 | 0.0625 | -0.506 |
| Between the DMN and SN | left middle occipital gyrus–right midcingulate cortex | 4.70 | < 0.0001 | 0.0010 | 0.583 |
|  | right cerebellar lobule IX–right middle frontal gyrus | 3.60 | 0.0008 | 0.0301 | -0.481 |

Abbreviations: AQ = Autism Spectrum Quotient; ASRS = Attention-Deficit Hyperactivity Disorder Self-Report Scale; FC = functional connectivity; Odd = oddball task; FPN = fronto-parietal network; DMN = default mode network; SN = salience network; df = degree of freedom; FDR = false discovery rate; β = standard partial regression coefficient

**Supplementary Table S3.** Network interactions that exhibit significant relationships between ASRS Inattention scores and FC values when ASRS Hyperactive score, age, and sex were used as covariates of no interest.

|  |  | **T-value**  **(df = 43)** | **Uncorrected *p*-value** | **FDR-adjusted *p*-value** | **β** |
| --- | --- | --- | --- | --- | --- |
| During Rest |  |  |  |  |  |
| Between the DMN and SN | right angular gyrus–right thalamus | -3.56 | 0.0009 | 0.0341 | -0.477 |

Abbreviations: ASRS = Attention-Deficit Hyperactivity Disorder Self-Report Scale; FC = functional connectivity; Odd = oddball task; DMN = default mode network; SN = salience network; df = degree of freedom; FDR = false discovery rate; β = standard partial regression coefficient

**Supplementary Table S4.** Network interactions that exhibit significant relationships between ASRS Hyperactive scores and FC values when ASRS Inattention score, age, and sex were used as covariates of no interest.

|  |  | **T-value**  **(df = 43)** | **Uncorrected *p*-value** | **FDR-adjusted *p*-value** | **β** |
| --- | --- | --- | --- | --- | --- |
| During Odd |  |  |  |  |  |
| Between the FPN and SN | left cingulate gyrus–right lobule VI | 3.23 | 0.0033 | 0.0499 | 0.442 |
|  | left superior parietal lobule–right lobule VI | 3.11 | 0.0033 | 0.0499 | 0.429 |
| Between the FPN and DMN | right gyrus frontalis superior pars medialis–right thalamus | 3.96 | 0.0003 | 0.0065 | 0.517 |
|  | left gyrus frontalis superior pars medialis-right thalamus | 3.68 | 0.0007 | 0.0065 | 0.489 |
|  | right cingulate gyrus–right thalamus | 3.68 | 0.0007 | 0.0065 | 0.489 |
| During Rest |  |  |  |  |  |
| Within the FPN | right gyrus frontalis superior pars medialis–right cingulate gyrus | -4.13 | 0.0002 | 0.0019 | -0.533 |
|  | right middle frontal gyrus–right middle temporal gyrus | -3.06 | 0.0038 | 0.0019 | -0.423 |

Abbreviations: ASRS = Attention-Deficit Hyperactivity Disorder Self-Report Scale; FC = functional connectivity; Odd = oddball task; DMN = default mode network; FPN = fronto-parietal network; SN = salience network; df = degree of freedom; FDR = false discovery rate; β = standard partial regression coefficient

**Supplementary Table S5.** Network interactions that exhibit significant relationships between AQ Social Skill scores and FC values when the scores of the other four AQ subscales, age, and sex were used as covariates of no interest.

|  |  | **T-value**  **(df = 40)** | **Uncorrected *p*-value** | **FDR-adjusted *p*-value** | **β** |
| --- | --- | --- | --- | --- | --- |
| During Odd |  |  |  |  |  |
| Within the SN | right lobule VI/crus I–left supramarginal gyrus/inferior parietal gyrus | -3.90 | 0.0004 | 0.0065 | -0.525 |
| Between the FPN and DMN | right gyrus frontalis superior pars medialis–right cerebellar lobule Ⅸ | -3.71 | 0.0006 | 0.0188 | -0.506 |
|  | left gyrus frontalis superior pars medialis–right cerebellar lobule Ⅸ | -3.27 | 0.0022 | 0.0337 | -0.459 |
| Between the SN and DMN | right cingulate gyrus–right thalamus | 3.68 | 0.0007 | 0.0065 | 0.503 |

Abbreviations: AQ = Autism Spectrum Quotient; FC = functional connectivity; Odd = oddball task; DMN = default mode network; FPN = fronto-parietal network; SN = salience network; df = degree of freedom; FDR = false discovery rate; β = standard partial regression coefficient

**Supplementary Table S6.** Network interactions that exhibit significant relationships between AQ Attention Switching scores and FC values when the scores of the other four AQ subscales, age, and sex were used as covariates of no interest.

|  |  | **T-value**  **(df = 40)** | **Uncorrected *p*-value** | **FDR-adjusted *p*-value** | **β** |
| --- | --- | --- | --- | --- | --- |
| During Odd |  |  |  |  |  |
| Within the FPN | right gyrus frontalis superior pars medialis–right middle frontal gyrus | -3.17 | 0.0030 | 0.0147 | -0.448 |
| Between the SN and DMN | right midcingulate cortex–left middle occipital gyrus | 3.98 | 0.0003 | 0.0184 | 0.533 |
|  | right supramarginal gyrus/inferior parietal gyrus–left middle occipital gyrus | 3.23 | 0.0025 | 0.0474 | 0.455 |
| During Rest |  |  |  |  |  |
| Within the FPN | right inferior parietal lobule–right middle frontal gyrus | 3.45 | 0.0013 | 0.0407 | 0.479 |
| Within the DMN | left parahippocampal gyrus–right parahippocampal gyrus | -3.54 | 0.0010 | 0.0430 | -0.488 |
|  | left parahippocampal gyrus–left middle occipital gyrus | -3.21 | 0.0026 | 0.0487 | -0.453 |
| Between the FPN and SN | right inferior parietal lobule–right midcingulate cortex | 3.20 | 0.0027 | 0.0325 | 0.451 |
|  | right inferior parietal lobule–right insula | 3.03 | 0.0043 | 0.0104 | 0.432 |
| Between the SN and DMN | right midcingulate cortex–left middle occipital gyrus | 3.34 | 0.0018 | 0.0459 | 0.467 |

Abbreviations: AQ = Autism Spectrum Quotient; FC = functional connectivity; Odd = oddball task; DMN = default mode network; FPN = fronto-parietal network; SN = salience network; df = degree of freedom; FDR = false discovery rate; β = standard partial regression coefficient

**Supplementary Table S7.** Network interactions that exhibit significant relationships between AQ Attention to Details scores and FC values when the scores of the other four AQ subscales, age, and sex were used as covariates of no interest.

|  |  | **T-value**  **(df = 40)** | **Uncorrected *p*-value** | **FDR-adjusted *p*-value** | **β** |
| --- | --- | --- | --- | --- | --- |
| During Odd |  |  |  |  |  |
| Within the FPN | right gyrus frontalis superior pars medialis–right middle temporal gyrus | -3.59 | 0.0009 | 0.0098 | -0.494 |
|  | left gyrus frontalis superior pars medialis–right middle temporal gyrus | -2.89 | 0.0062 | 0.0339 | -0.416 |
| During Rest |  |  |  |  |  |
| Within the SN | right lobule VI/crus I–left lobule VI/crus I | -3.45 | 0.0013 | 0.0237 | -0.479 |

Abbreviations: AQ = Autism Spectrum Quotient; FC = functional connectivity; Odd = oddball task; FPN = fronto-parietal network; SN = salience network; FDR = false discovery rate; df = degree of freedom; β = standard partial regression coefficient

**Supplementary Table S8.** Network interactions that exhibit significant relationships between AQ Communication scores and FC values when the scores of the other four AQ subscales, age, and sex were used as covariates of no interest.

|  |  | **T-value**  **(df = 40)** | **Uncorrected *p*-value** | **FDR-adjusted *p*-value** | **β** |
| --- | --- | --- | --- | --- | --- |
| During Rest |  |  |  |  |  |
| Within the FPN | left middle temporal gyrus–right gyrus frontalis superior pars medialis | 3.02 | 0.0044 | 0.0488 | 0.431 |

Abbreviations: AQ = Autism Spectrum Quotient; FC = functional connectivity; FPN = fronto-parietal network; df = degree of freedom; FDR = false discovery rate; β = standard partial regression coefficient

**Supplementary Table S9.** Network interactions that exhibit significant relationships between AQ Imagination scores and FC values when the scores of the other four AQ subscales, age, and sex were used as covariates of no interest.

|  |  | **T-value**  **(df = 40)** | **Uncorrected *p*-value** | **FDR-adjusted *p*-value** | **β** |
| --- | --- | --- | --- | --- | --- |
| During Odd |  |  |  |  |  |
| Within the SN | left supramarginal gyrus/inferior parietal gyrus–right lobule VI/crus I | 3.78 | 0.0005 | 0.0093 | 0.513 |
|  | left posterior insula/putamen–left middle frontal gyrus | 3.19 | 0.0028 | 0.0259 | 0.450 |
|  | right midcingulate cortex–left middle frontal gyrus | 3.18 | 0.0029 | 0.0259 | 0.449 |
|  | right midcingulate cortex–left insula | 2.94 | 0/0054 | 0.0484 | 0.422 |
| Within the DMN | left parahippocampal gyrus–right posterior cingulate cortex/precuneus | -3.54 | 0.0041 | 0.04 | -0.488 |
|  | left hippocampus–right posterior cingulate cortex/precuneus | 2.95 | 0.0053 | 0.0480 | 0.423 |
| Between the SN and DMN | left posterior insula/putamen–right superior frontal gyrus | 3.34 | 0.0018 | 0.0487 | 0.467 |
| During Rest |  |  |  |  |  |
| Within the DMN | left hippocampus–right superior frontal gyrus/middle frontal gyrus | 3.48 | 0.0012 | 0.0176 | 0.482 |
|  | right hippocampus–right angular gyrus | 3.45 | 0.0013 | 0.0233 | 0.479 |
|  | left hippocampus–left parahippocampal cortex | 3.32 | 0.0020 | 0.0176 | 0.465 |
|  | left hippocampus–right angular gyrus | 2.78 | 0.0082 | 0.0495 | 0.402 |
| Between the FPN and SN | right cingulate gyrus–left insula | 3.47 | 0.0013 | 0.0191 | 0.481 |
|  | right gyrus frontalis superior pars medialis–left insula | 3.47 | 0.0013 | 0.0191 | 0.481 |
| Between the SN and DMN | right supramarginal gyrus/inferior parietal gyrus–left parahippocampal cortex | -3.80 | 0.0005 | 0.0179 | -0.515 |
|  | right supramarginal gyrus/inferior parietal gyrus–right cerebellar lobule Ⅸ | -3.51 | 0.0011 | 0.0362 | -0.485 |

Abbreviations: AQ = Autism Spectrum Quotient; FC = functional connectivity; Odd = oddball task; DMN = default mode network; FPN = fronto-parietal network; SN = salience network; df = degree of freedom; FDR = false discovery rate; β = standard partial regression coefficient

**Supplementary Table S10.** Network interactions that exhibit significant relationships of both AQ and ASRS total scores with FC values when age and sex were used as covariates of no interest.

|  |  | **T-value**  **(df = 43)** | **Uncorrected *p*-value** | **FDR-adjusted *p*-value** | **β** |
| --- | --- | --- | --- | --- | --- |
| During Rest |  |  |  |  |  |
| Between the DMN and SN | left middle occipital gyrus–right midcingulate cortex | 4.32 | 0.0001 | 0.0044 | 0.550 |

Abbreviations: AQ = Autism Spectrum Quotient; ASRS = Attention-Deficit Hyperactivity Disorder Self-Report Scale; FC = functional connectivity; Odd = oddball task; DMN = default mode network; FPN = fronto-parietal network; SN = salience network; df = degree of freedom; FDR = false discovery rate; β = standard partial regression coefficient

**Supplementary Table S11.** Network interactions that exhibit significant correlations between ASRS scores and FC values within Group 1.

|  |  | **T-value**  **(df = 20)** | **Uncorrected *p*-value** | **FDR-adjusted *p*-value** | **β** |
| --- | --- | --- | --- | --- | --- |
| During Odd |  |  |  |  |  |
| Within the SN | right supramarginal gyrus/inferior parietal gyrus–right thalamus | 3.13 | 0.0053 | 0.0477 | 0.573 |
|  | right supramarginal gyrus/inferior parietal gyrus–left supramarginal gyrus/inferior parietal gyrus | 3.06 | 0.0053 | 0.0477 | 0.565 |
| Between the DMN and SN | left hippocampus–right lobule VI | -4.34 | 0.0003 | 0.0117 | -0.696 |
| During Rest |  |  |  |  |  |
| Between the DMN and FPN | right superior frontal gyrus–left middle frontal gyrus | 4.43 | 0.0003 | 0.0078 | 0.704 |

Abbreviations: ASRS = Attention-Deficit Hyperactivity Disorder Self-Report Scale; FC = functional connectivity; Odd = oddball task; DMN = default mode network; FPN = fronto-parietal network; SN = salience network; df = degree of freedom; FDR = false discovery rate; β = standard partial regression coefficient

**Supplementary Table S12.** Network interactions that exhibit significant correlations between ASRS scores and FC values within Group 2.

|  |  | **T-value**  **(df = 20)** | **Uncorrected *p*-value** | **FDR-adjusted *p*-value** | **β** |
| --- | --- | --- | --- | --- | --- |
| During Odd |  |  |  |  |  |
| Within the DMN | right angular gyrus/middle occipital gyrus–right superior frontal gyrus/middle frontal gyrus | 4.08 | 0.0006 | 0.0106 | 0.674 |
| During Rest |  |  |  |  |  |
| Within the DMN | right angular gyrus/middle occipital gyrus–right superior frontal gyrus/middle frontal gyrus | 3.53 | 0.0021 | 0.0374 | 0.620 |

Abbreviations: ASRS = Attention-Deficit Hyperactivity Disorder Self-Report Scale; FC = functional connectivity; Odd = oddball task; DMN = default mode network; df = degree of freedom; FDR = false discovery rate; β = standard partial regression coefficient

**Supplementary Table S13.** Network interactions that exhibit significant correlations between AQ scores and FC values within Group 1.

|  |  | **T-value**  **(df = 20)** | **Uncorrected *p*-value** | **FDR-adjusted *p*-value** | **β** |
| --- | --- | --- | --- | --- | --- |
| During Odd |  |  |  |  |  |
| Within the SN | left lobule VI/crus I–left middle frontal gyrus | 3.68 | 0.0015 | 0.0264 | 0.635 |
|  | left middle occipital gyrus–left posterior insula/putamen | -3 | 0.0402 | 0.0439 | -0.557 |
| During Rest |  |  |  |  |  |
| Within the FPN | left inferior parietal lobe–left middle frontal gyrus | 3.72 | 0.0013 | 0.0147 | 0.639 |
| Between the FPN and SN | right superior parietal lobule–left posterior insula/putamen | 4.15 | 0.0005 | 0.0150 | 0.680 |
|  | left superior parietal lobule–left posterior insula/putamen | 3.78 | 0.0012 | 0.0269 | 0.646 |
|  | left superior parietal lobule–right midcingulate cortex | 3.60 | 0.0018 | 0.0269 | 0.627 |
|  | right superior parietal lobule–right midcingulate cortex | 3.42 | 0.0027 | 0.0411 | 0.607 |

Abbreviations: AQ = Autism Spectrum Quotient; FC = functional connectivity; Odd = oddball task; FPN = fronto-parietal network; SN = salience network; df = degree of freedom; FDR = false discovery rate; β = standard partial regression coefficient

**Supplementary Table S14.** Network interactions that exhibit significant correlations between AQ scores and FC values within Group 2.

|  |  | **T-value** | **Uncorrected *p*-value** | **FDR-adjusted *p*-value** | **β** |
| --- | --- | --- | --- | --- | --- |
| During Rest |  |  |  |  |  |
| Within the DMN | right superior frontal gyrus/middle frontal gyrus–right angular gyrus/middle occipital gyrus | 3.58 | 0.0019 | 0.0336 | 0.625 |
| Between the FPN and DMN | right inferior parietal lobule–right angular gyrus/middle occipital gyrus | 4.43 | 0.0003 | 0.0077 | 0.704 |
| Between the FPN and SN | left middle temporal gyrus–right midcingulate cortex | 4.15 | 0.0005 | 0.0148 | 0.680 |
|  | left superior parietal lobule–right cerebellar lobule VI | -3.87 | 0.0009 | 0.0284 | -0.654 |
| Between the DMN and SN | right retro–splenial cortex/posterior cingulate cortex–right midcingulate cortex | 4.13 | 0.0005 | 0.0097 | 0.678 |
|  | left middle occipital gyrus-right midcingulate cortex | 5.97 | <0.0001 | 0.0003 | 0.800 |

Abbreviations: AQ = Autism Spectrum Quotient; FC = functional connectivity; DMN = default mode network; FPN = fronto-parietal network; SN = salience network; df = degree of freedom; FDR = false discovery rate; β = standard partial regression coefficient

**Supplementary Table 15.** The network interactions that exhibited significant correlations between the ADHD-RS scores and FC values within the ADHD-200 data set.

|  |  | **T-value**  **(df=43)** | **Uncorrected *p*-value** | **FDR-adjusted *p*-value** | **β** |
| --- | --- | --- | --- | --- | --- |
| During Rest |  |  |  |  |  |
| Within the SN | left lobule VI/crus I–left lobule VI | 3.20 | 0.0026 | 0.0465 | 0.439 |
| Between the DMN and FPN | right parahippocampal gyrus–right gyrus frontalis superior pars medialis | 3.19 | 0.0027 | 0.0403 | 0.437 |
|  | right parahippocampal gyrus–right cingulate gyrus | 3.19 | 0.0027 | 0.0403 | 0.437 |

Abbreviations: ADHD-RS = attention-deficit hyperactivity disorder rating scale; Odd = the oddball task; DMN = default mode network; FPN = fronto-parietal network; SN = salience network; df = degree of freedom; FDR = false discovery rate; β= standard partial regression coefficient

**Supplementary Table 16.** The network interactions that exhibited significant relationships between ADHD-RS Hyperactivity scores and FC values within the ADHD-200 data set.

|  |  | **T-value**  **(df=43)** | **Uncorrected *p*-value** | **FDR-adjusted *p*-value** | **β** |
| --- | --- | --- | --- | --- | --- |
| During Rest |  |  |  |  |  |
| Within the FPN | right middle temporal gyrus–left middle frontal gyrus | 3.02 | 0.0043 | 0.047 | 0.418 |
| Within the SN | left lobule VI/crus I–left lobule VI | 3.30 | 0.0020 | 0.0354 | 0.450 |

Abbreviations: ADHD-RS = attention-deficit hyperactivity disorder rating scale; Odd = the oddball task; FPN = fronto-parietal network; SN = salience network. FDR = False Discovery Rate

**Supplementary Table 17.** The network interactions that exhibited significant correlations between the SRS scores and FC values within the ABIDE data set.

|  |  | **T-value**  **(df=34)** | **Uncorrected *p*-value** | **FDR-adjusted *p*-value** | **β** |
| --- | --- | --- | --- | --- | --- |
| During Rest |  |  |  |  |  |
| Within the DMN | right cerebellar lobule VI–left angular gyrus | -3.43 | 0.0016 | 0.0292 | -0.507 |
|  | right cerebellar lobule VI–medial prefrontal cortex/anterior cingulate cortex/orbitofrontal cortex | -3.01 | 0.0050 | 0.0446 | -0.459 |
| Within the FPN | left gyrus frontalis superior pars medialis-right gyrus frontalis superior pars medialis | -3.16 | 0.0033 | 0.018 | -0.476 |
|  | left gyrus frontalis superior pars medialis–right cingulate gyrus | -3.16 | 0.0033 | 0.018 | -0.476 |
| Between the DMN and FPN | left angular gyrus–left gyrus frontalis superior pars medialis | 3.01 | 0.0049 | 0.0487 | 0.459 |
| Between the DMN and SN | left parahippocampal corte–right superior parietal gyrus/precuneus | 3.37 | 0.0019 | 0.0453 | 0.500 |
|  | Precuneus–right superior parietal gyrus/precuneus | 3.27 | 0.0024 | 0.0453 | 0.489 |
|  | left angular gyrus–right lobule VI | -3.28 | 0.0024 | 0.0450 | -0.490 |

Abbreviations: SRS = Social Responsiveness Scale; Odd = the oddball task; DMN = default mode network; FPN = fronto-parietal network; SN = salience network; df = degree of freedom; FDR = false discovery rate; β= standard partial regression coefficient

**Supplementary Table S18.** Network interactions that exhibit significant correlations between ASRS scores and FC values using ROIs from the Harvard-Oxford Atlas.

|  |  | **T-value**  **(df = 44)** | **Uncorrected *p*-value** | **FDR-adjusted *p*-value** | **β** |
| --- | --- | --- | --- | --- | --- |
| During Rest |  |  |  |  |  |
| Within the DMN | right lateral parietal cortex–precuneus | -2.82 | 0.0073 | 0.00218 | -0.391 |

Abbreviations: ASRS = Attention-Deficit Hyperactivity Disorder Self-Report Scale; FC = functional connectivity; ROI = region of interest; Odd = oddball task; DMN = default mode network; df = degree of freedom; FDR = false discovery rate; β = standard partial regression coefficient

**Supplementary Table S19.** Network interactions that exhibit significant correlations between AQ Total scores and FC values using ROIs from the Harvard-Oxford Atlas.

|  |  | **T-value**  **(df = 44)** | **Uncorrected *p*-value** | **FDR-adjusted *p*-value** | **β** |
| --- | --- | --- | --- | --- | --- |
| During Odd |  |  |  |  |  |
| Within the FPN | left lateral prefrontal cortex lobe–right lateral prefrontal cortex | 2.73 | 0.0092 | 0.0275 | 0.381 |
| Between the FPN and DMN | left lateral prefrontal cortex lobe–right lateral parietal cortex | 2.62 | 0.0120 | 0.0419 | 0.367 |
| Between the FPN and SN | left lateral prefrontal cortex lobe–right anterior insula | -3.16 | 0.0028 | 0.0423 | -0.430 |
| During Rest |  |  |  |  | 0.000 |
| Within the FPN | left lateral prefrontal cortex lobe–right lateral prefrontal cortex lobe | 2.62 | 0.0120 | 0.0360 | 0.367 |
|  | left posterior parietal cortex–right lateral prefrontal cortex lobe | 2.46 | 0.0120 | 0.0360 | 0.348 |

Abbreviations: AQ = Autism Spectrum Quotient; FC = functional connectivity; ROI = region of interest; Odd = oddball task; DMN = default mode network; FPN = fronto-parietal network; SN = salience network; df = degree of freedom; FDR = false discovery rate; β = standard partial regression coefficient

**Supplementary Table S20.** Network interactions that exhibit significant correlations between ASRS scores and FC values.

|  |  | **T-value**  **(df=44)** | **Split-half**  **Group 1:**  **T-value (df=20)/FDR-adjusted *p*-value** | **Split-half**  **Group 2:**  **T-value (df=20)/ FDR-adjusted *p*-value** |
| --- | --- | --- | --- | --- |
| During Odd |  |  |  |  |
| Within the DMN | right angular gyrus–thalamus | 3.47 | 2.25/ 0.6477 | 2.08/ 0.3049 |
|  | thalamus–right superior frontal gyrus and middle frontal gyrus | 3.06 | 1.43/0.1668 | 2.10/ 0.2861 |
| Between the FPN and SN | right gyrus frontalis superior pars medialis–right lobule VI(CBM) | 3.12 | 2.92/ 0.1182 | 1.62/ 0.5790 |
|  | right cingulate gyrus–right lobule VI(CBM) | 3.12 | 2.15/ 0.4938 | 1.62/ 0.5790 |

Abbreviations: ASRS = Attention-Deficit Hyperactivity Disorder Self-Report Scale; FC = functional connectivity; Odd = oddball task; DMN = default mode network; FPN = fronto-parietal network; SN = salience network; CBM = cerebellum; df = degree of freedom; FDR = false discovery rate

**Supplementary Table S21.** Network interactions that exhibit significant correlations between AQ scores and FC values.

|  |  | **T-value**  **(df=44)** | **Split-half**  **Group 1:**  **T-value**  **(df=20)/FDR-adjusted *p*-value** | **Split-half**  **Group 2:**  **T-value**  **(df=20)/FDR-adjusted *p*-value** | **ABIDE dataset:**  **T-value**  **(df=34)/FDR-adjusted *p*-value** |
| --- | --- | --- | --- | --- | --- |
| During Odd |  |  |  |  |  |
| Within the FPN | left inferior parietal lobe–right inferior parietal lobe | 3.41 | 1.88/ 0.2983 | 2.37/0.2540- | - |
|  | left medial frontal gyrus–right inferior parietal lobe | 2.91 | 2.34/ 0.1902 | 0.79/0.6004 | - |
| Between the FPN and SN | left inferior parietal lobe –right posterior insula | -3.16 | -1.66/ 0.5293 | -2.20/ 0.1790 | - |
|  | left inferior parietal lobe–left posterior insula/putamen | -3 | -1.75/ 0.4694 | -2.50/ 0.1790 | - |
| During Rest |  |  |  |  |  |
| Within the SN | left lobule VI/crus I (CBM)–right lobule VI/crus I (CBM) | -3.3 | 0.87/ 0.7848 | -1.29/ 0.3285 | -1.36/0.3873 |
| Between the DMN and SN | left middle occipital gyrus–right midcingulate cortex | 5 | -0.24/0.9590 | 2.22/0.3498 | -1.51/0.8278 |
|  | right retro–splenial cortex/posterior cingulate cortex–right midcingulate cortex | 3.23 | 1.60/ 0.9418 | 4.13/0.0097* | -0.49/0.9506 |

Abbreviations: AQ = Autism-Spectrum Quotient; FC = functional connectivity; Odd = oddball task; DMN = default mode network; FPN = fronto-parietal network; SN = salience network; CBM = cerebellum; df = degree of freedom; FDR = false discovery rate
